# Characterization of the Jomthonic Acids Biosynthesis Pathway and Isolation of Novel Analogues in *Streptomyces caniferus* GUA-06-05-006A

**DOI:** 10.3390/md16080259

**Published:** 2018-07-31

**Authors:** Raúl García-Salcedo, Rubén Álvarez-Álvarez, Carlos Olano, Librada Cañedo, Alfredo F. Braña, Carmen Méndez, Fernando de la Calle, José A. Salas

**Affiliations:** 1 Department of Functional Biology and University Institute of Oncology of Principado de Asturias (U.I.O.P.A), University of Oviedo, 33006 Oviedo (Asturias), Spain; raul.garcia.salcedo@gmx.com (R.G.-S.); ruben.alvarez.microbio@gmail.com (R.Á.-Á); olanocarlos@uniovi.es (C.O.); afb@uniovi.es (A.F.B.); cmendezf@uniovi.es (C.M.); 2 Institute for Health Research of Principado de Asturias (IHRPA), 33006 Oviedo (Asturias), Spain; 3 Drug Discovery Area, PharmaMar S.A. Avda. de los Reyes 1, 28770 Colmenar Viejo (Madrid), Spain; lcanedo@pharmamar.com (L.C.); fdelacalle@pharmamar.com (F.d.l.C.)

**Keywords:** biosynthesis gene cluster, molecular elicitation, heterologous expression, polyketide synthase, nonribosomal peptide synthetase

## Abstract

Jomthonic acids (JAs) are a group of natural products (NPs) with adipogenic activity. Structurally, JAs are formed by a modified β-methylphenylalanine residue, whose biosynthesis involves a methyltransferase that in *Streptomyces hygroscopicus* has been identified as MppJ. Up to date, three JA members (A–C) and a few other natural products containing β-methylphenylalanine have been discovered from soil-derived microorganisms. Herein, we report the identification of a gene (*jomM*) coding for a putative methyltransferase highly identical to MppJ in the chromosome of the marine actinobacteria *Streptomyces caniferus* GUA-06-05-006A. In its 5’ region, *jomM* clusters with two polyketide synthases (PKS) (*jomP1*, *jomP2*), a nonribosomal peptide synthetase (NRPS) (*jomN*) and a thioesterase gene (*jomT*), possibly conforming a single transcriptional unit. Insertion of a strong constitutive promoter upstream of *jom*P1 led to the detection of JA A, along with at least two novel JA family members (D and E). Independent inactivation of *jomP1*, *jomN* and *jomM* abolished production of JA A, JA D and JA E, indicating the involvement of these genes in JA biosynthesis. Heterologous expression of the JA biosynthesis cluster in *Streptomyces coelicolor* M1152 and in *Streptomyces albus* J1074 led to the production of JA A, B, C and F. We propose a pathway for JAs biosynthesis based on the findings here described.

## 1. Introduction

The marine environment is a valuable source of natural products (NPs) with increasing medical importance [1,2]. The relevance and success of screening programs aimed at discovering new molecules with pharmaceutical application from the marine environment has been validated by the development and marketing of various NPs as analgesic [3], antiviral [4] and antitumor drugs [5]. This fact, along with the exponentially growing number of reported bioactive agents isolated from marine invertebrates and microorganisms [6,7], points to oceans as a preeminent environment for the supply of novel therapeutic chemicals. Although marine invertebrates such as sponges [8,9] are renowned drug discovery targets, in many instances the true producer of the relevant NP are symbiotic microorganisms, often bacteria and cyanobacteria, which represent a worthy exploring source of novel and structurally diverse chemical entities [10,11,12,13].

In the last decade, much attention has been given to microbial genomics regarding the potential of microorganisms as producers of bioactive metabolites [14,15,16]. Many whole-genome sequencing projects from both marine- and soil-derived microorganisms have uncovered a wealth of orphan genes related to secondary metabolism such as polyketide synthases (PKS) and non-ribosomal peptide synthetases (NRPS) [17,18]. This way, the blooming number of microbial genomes and metagenomic data from different environments [19,20,21] might contribute to innovative achievements on NP research and raises new prospects for the discovery of novel treatments for emerging disorders [22,23,24]. Genomic analysis of known and under study microorganisms reveals that the number of potential secondary metabolite gene clusters harbored by their respective chromosomes surpasses the amount of compounds detected under conventional laboratory conditions, indicating that microbial capability to provide chemicals of medical interest could be far greater than expected [25,26,27]. This has prompted the development of a series of molecular- and cultivation-based approaches intended to uncover and exploit the natural chemical potential of candidate microorganisms by eliciting the expression of silent or poorly expressed gene clusters [28,29,30,31,32]. Alone or in combination, different pathway-specific genome mining strategies such as gene mutation, insertion of constitutive promoters in front of key gene operons or direct cloning and subsequent heterologous expression of orphan gene clusters, might help to uncover the metabolite(s) biosynthesized by any given cryptic gene cluster. These and many other molecular strategies, along with the development of next-generation whole-genome sequencing systems and bioinformatics sequence analysis platforms, have given birth to the so-called field of genomics-driven natural product discovery [24,33,34]. Research on this field has made feasible the discovery of a wave of novel microbial NPs biosynthesized by gene clusters regarded up to now as cryptic [35,36,37,38,39,40,41].

Herein, we report on the activation of a cryptic PKS-NRPS hybrid gene cluster in the chromosome of the marine actinobacteria *Streptomyces caniferus* GUA-06-05-006A. This has enabled the identification of three novel metabolites belonging to the jomthonic acid (JA) family of NPs. Up to date, only three members (JA A–C) of this family of molecules have been reported [42,43]. We describe the isolation and structural determination of two new JA A derivatives, and the identification of additional potential related compounds.

## 2. Results

### 2.1. Identification of the Jomthonic Acid Gene Cluster

**Figure 1:**
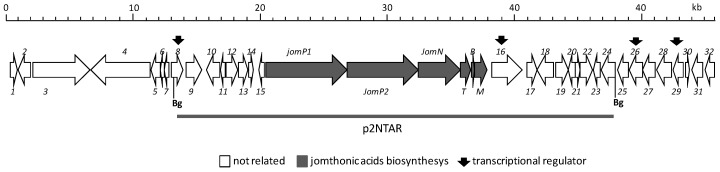
Organization of the jomthonic acids biosynthetic gene cluster. The proposed gene functions are listed in Table 1. Grey bar indicates DNA fragment cloned in plasmid pJATAR for heterologous expression.

**Figure 2:**
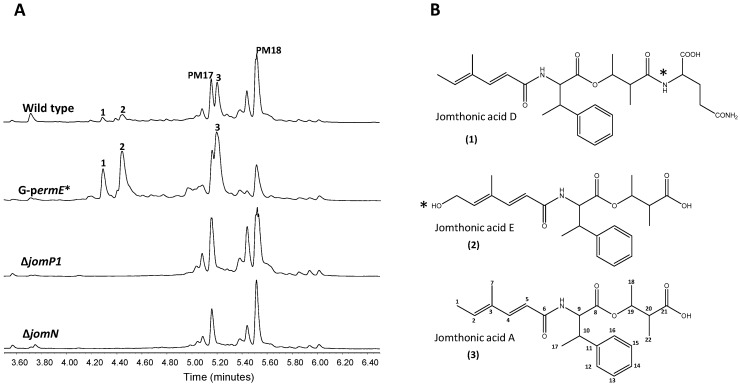
Activation of jomthonic acids biosynthesis. (**A**) Metabolite analysis by UPLC of *Streptomyces caniferus* GUA-06-05-006A (wild type), G-perm*strain (constitutive promoter ermE*p inserted) and mutants Δ*jomP1* and Δ*jomN*. Peaks corresponding to PM100117 (PM17) and PM100118 (PM18) are indicated. (**B**) Chemical structures of jomthonic acid A and novel derivatives D and E. Asterisks indicate structural differences among the three compounds.

Previous identification and characterization in the marine actinobacteria *S. caniferus* GUA-06-05-006A of the promising antitumor compounds PM100117 and PM100118 biosynthesis gene cluster (BGC) [44], aimed us to further investigate the chemical diversity enclosed in this strain. Of the 32 BGCs potentially involved in the production of secondary metabolites detected after sequencing of the genome of *S. caniferus* GUA-06-05-006A, only two BGCs contain PKS–NRPS hybrid genes [44]. One of these PKS–NRPS BGCs (Figure 1, Table 1) caught our attention because it entails a large operon that included a gene (*jomM*) coding for a putative methyltransferase highly identical to MppJ, the enzyme responsible for the biosynthesis of βMePhe in *Streptomyces hygroscopicus* [45]. To discover the compound(s) coded by this BGC, we inserted the constitutive promoter ermE*p upstream of the PKS gene *jomP1* (Figure 1). Ultra Performance Liquid Chromatography (UPLC) analysis of metabolite production in the resulting strain, G-permE*, revealed the increased biosynthesis, relative to wild type, of three compounds (**1**–**3**, Figure 2A) showing identical UV spectra, with a maximum absorption wavelength at 264 nm. Consistent with this observation, production of **1**–**3** was abolished upon insertional inactivation of PKS gene *jomP1* or NRPS gene *jomN* (Figure 2A), thus confirming the involvement of both genes in **1**–**3** biosynthesis. These compounds were purified and subjected to structural analysis by nuclear magnetic resonance NMR (Supplementary file 1). The chemical structure of **1** and **2** corresponded to novel analogues of JA A, a previously identified natural product with adipogenic activity produced by *Streptomyces* sp. BB47 [42]. Structurally, JA A (peak **3**, UPLC *R_t_* = 5.209 min, *m*/*z* 388.2141[*M* + H]^+^ (calculated for C_22_H_30_NO_5_ 388.2118)), consists of a βMePhe residue with its amino and carboxyl group attached to a short polyketide chain and to a hydroxyacid unit, respectively (Figure 2B). The chemical structure of **1** (UPLC *R_t_* = 4,264 min, *m*/*z* 516.2709 [*M* + H]^+^ (calculated for C_27_H_38_N_3_O_7_ 516.2704)) is that of JA A with a glutamine residue at C21 in the hydroxyacid part. Compound **2** (UPLC *R_t_* = 4,411 min, *m*/*z* 404.2099 [*M* + H]^+^ (calculated for C_22_H_30_NO_6_ 404. 2079)) is derived from **3** by polyketide chain hydroxylation at C1. Given the structural relationship of compounds **1** and **2** with JA A, hereinafter, they will be referred to as JA D and E, respectively.

### 2.2. Bioinformatics Analysis of the Jomthonic Acid Gene Cluster and Proposed Biosynthesis Pathway

**Figure 3:**
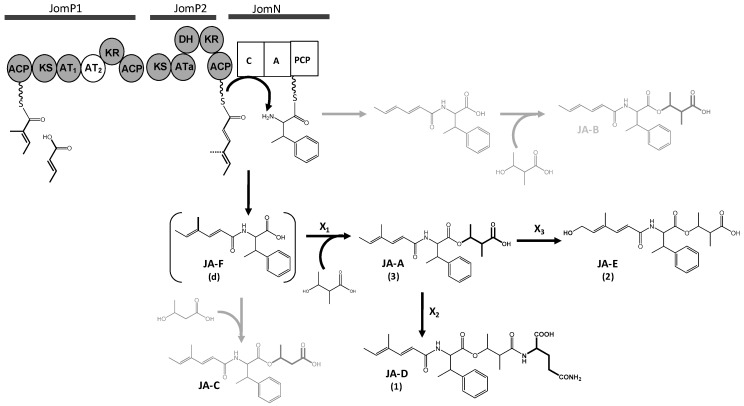
Proposed biosynthesis pathway of jomthonic acids. Polyketide synthase (*JomP1* and *JomP2*) and non-ribosomal protein synthetase (*JomN*) domains are as follows: KS ketosynthase, AT acyltransferase, KR ketoreductase, DH dehydratase, ACP acyl-carrier-protein, C condensation, Aadenylation, PCP peptidyl carrier protein. Compounds not detected in *S. caniferus* GUA-06-05-006A are shown in grey. Parentheses indicate structures not confirmed by NMR. Genes coding for enzymes X_1_, X_2_ and X_3_ were not identified in the biosynthesis gene cluster.

*Polyketide biosynthesis*: The predicted JAs BGC spans 55kb and contains 38 ORFs, which includes two putative PKS genes, *jomP1* and *jomP2* (Figure 1 and Table 1), presumably involved in the biosynthesis of the JA polyketide chain. JomP1 and JomP2 show the closest similarity with PKS proteins StiA (57%) and StiC (49%), respectively, which participate in the biosynthesis of stigmatellin in the myxobacteria *Stigmatella aurantica* Sga15 [46]. Besides, both proteins show resemblance with putative PKS enzymes from other myxobacteria members and several species of cyanobacteria. Bioinformatics analysis detected in JomP1 an uncommon PKS domain pattern, consisting of two ACP and two contiguous AT (AT_1_ and AT_2_) domains, distributed as follows: ACP-KS-AT_1_-AT_2_-KR-ACP (Figure 3). This pattern is a rare finding previously described in myxobacteria [46,47,48,49]. In these microorganisms, the presence of two adjacent AT domains has been linked to the specificity of the loading domain for unusual initiating units, alternative to malonyl- and methylmalonyl-CoA [46]. It has been conjectured that in the biosynthesis of myxalamid [48], soraphen [49] and myxothiazol [47], the first AT domain is responsible for loading the unusual starter units, isobutyryl-CoA/2-methyl-butyryl-CoA, benzoyl-CoA and methylbutyryl-CoA, respectively, while the second AT domain is involved in the transfer of the first extension unit, typically malonyl- or methylmalonyl-CoA. However, this assumption might not stand for the JomP1 AT_2_ domain, which might be inactive due to the substitution of Ser-7 and Gln-8 for Gly and His, respectively, in the conserved sequence domain GqgSQ (Supplementary file 2: Figure S22) that lies close to the active site pocket in the AT domain 3D model [50,51]. According to these observations, it seems reasonable to propose the transfer by AT_1_ of 2-methyl-2-eno-butyryl-CoA (MEB-CoA) as the carboxylic acid incorporated by JomP1 (Figure 3). Furthermore, a certain degree of flexibility of AT_1_ could explain the biosynthesis of other JAs previously described [42,43]. In this hypothetical model, no role can be envisaged for the JomP1 KR domain. The biosynthesis of the JAs polyketide chain is completed by PKS JomP2, which contains an AT domain with predicted specificity for malonyl-CoA, KR and DH domains.

**Figure 4:**
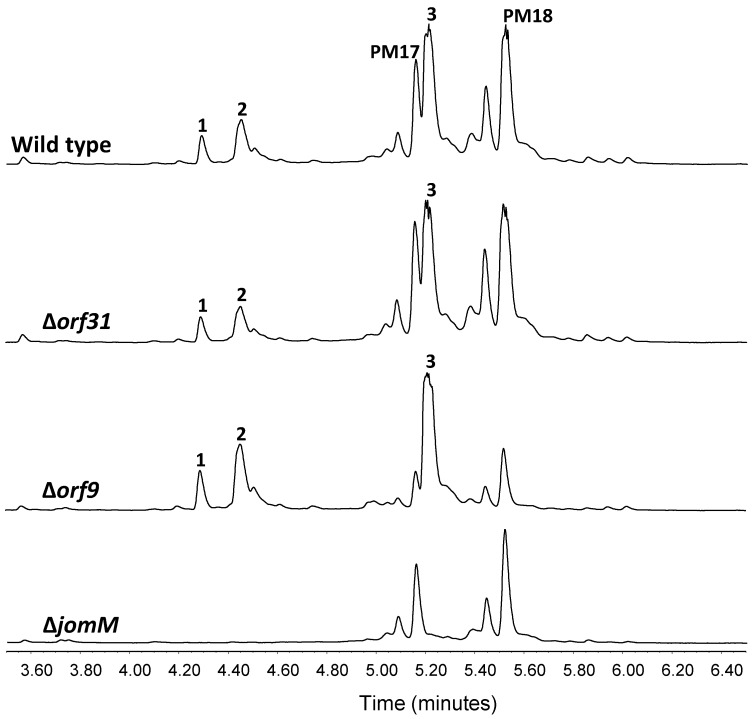
Deletion of *jomM* and genes potentially involved in JA D and E biosynthesis. UPLC analysis of jomthonic acids biosynthesis in *Streptomyces caniferus* GUA-06-05-006A (wild type) and mutant strains ∆*orf31*, ∆*orf9* and ∆*jomM*. Peaks corresponding to PM100117 (PM17) and PM100118 (PM18) are indicated.

*Methylphenylalanine biosynthesis*: A (2S, 3R) βMePhe residue constitutes the structural core of JAs. Biosynthesis of this moiety presumably requires activity of a methyltransferase enzyme. Genetic experiments in *Streptomyces hygroscopicus* NRRL30439 have shown methyltransferase MppJ to catalyze phenylalanine β-methylation in mannopeptimycin biosynthesis [45]. Besides, later in vitro studies on MppJ activity have shown that it methylates the benzylic C atom of phenylpyruvate instead of phenylalanine or the mannopeptimycin aglycone F [52]. Gene *jomM* in the JAs BGC codes for a putative methyltranferase 55% identical to MppJ. UPLC analysis of culture extracts from deletion mutant ∆*jomM* showed loss of **1**–**3** production (Figure 4), confirming involvement in JAs biosynthesis, conceivably catalyzing phenylalanine β-methylation. JA biosynthesis was restored in ∆*jomM* upon *jomM* re-introduction (Supplementary file 2: Figure S23).

*2-methyl-3-hydroxybutyrate biosynthesis and other gene functions*: Various branched-chain fatty acids, such as 2-methylbutyrate, can be generated in bacteria from amino acid fermentation. Moreover, interconversion between 2-methy-3-hydroxylbutyrate (MHB) and leucine has been described in anaerobic bacteria [53,54]. Hence, although based on sequence identity, no gene functions remotely related to MHB biosynthesis are comprised within the JA BGC; certain primary metabolism reactions might supply this structural moiety.

The JA BGC also comprises NRPS *jomN*, containing a condensation and an adenylation domain, which is postulated to be the candidate enzyme to catalyze βMePhecondensation with the polyketide chain. MbtH-like protein JomB and thioesterase JomT complete the proteins presumably required for JAs biosynthesis. Based on various studies on MbtH-like protein function [55,56], JomB is possibly required for βMePhe activation during its adenylation by JomN. JomT might be required to cleave and offload JA F from the JomN PCP domain (Figure 3).

*Hypothetical model for JAs assembly*: Polyketide biosynthesis might be commenced by JomP1 which, despite containing two ACP and two AT domains, would only be responsible for the loading of the starting carboxylic acid MEB (Figure 3). As explained above, no extension reaction is presumed to be catalyzed by JomP1 AT_2_. JomP2 would then extend the polyketide chain with malonate, with a double bond formation due to the consecutive activity of its KR and DH domain. Next, NRPS JomN would perform condensation of βMePhe with the polyketide molecule to form JA F. Subsequent addition of MHB to form JA A might require JA F release from JomN, a reaction probably catalyzed by JomT. Notwithstanding, the enzymatic activity (X_1_) required to transfer the third JAs structural moiety MHB to JA F (Figure 3) is unknown and possibly coded elsewhere in the genome as, based on sequence identity, no gene functions suited to accomplish this reaction have been identified within the JA BGC. Likewise, the JA BGC does not code for enzymatic activities related to glutamine transfer (X_2_) to produce JA D and JA hydroxylation (X_3_) to produce JA E (Figure 3). Gene candidates conceivably involved is those reaction are *orf31* and *orf9*, which encode for putative proteins with glutamine binding lipoprotein and monooxygenase activity, respectively. Nevertheless, deletion of these genes does not have any effect on JA D and E production (Figure 4).

### 2.3. Cluster Boundaries Analysis

**Figure 5:**
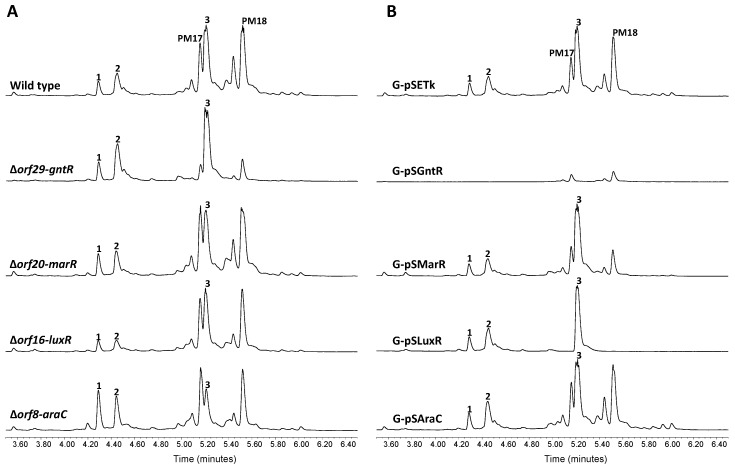
Cluster boundaries analysis. UPLC analysis of jomthonic acids production in (**A**) *Streptomyces caniferus* GUA-06-05-006A (wild type) and mutant strains ∆*orf29-gntR*, ∆*orf20-marR*, ∆*orf16-luxR* and ∆*orf8-araC*, and (**B**) *Streptomyces caniferus* GUA-06-05-006A carrying overexpression plasmids pSETk (empty plasmid), pSEGntR (*orf29*), pSEMarR (*orf20*), pSESARP (*orf16*) and pSEAraC (*orf8*). Peaks corresponding to PM100117 (PM17) and PM100118 (PM18) are indicated.

Flanking the JAs BGC there are four *orfs* coding for putative transcriptional regulators belonging to the GntR (*orf29*), MarR (*orf20*), LuxR (*orf16*) and AraC (*orf8*) families of transcription factors. To examine whether or not these putative regulators take part in JAs biosynthesis control, we generated a series of single gene deletion strains. UPLC metabolite analysis of the resulting mutants ∆*orf29-gntR*, ∆*orf20-marR* and ∆*orf16-luxR*, revealed no significant alteration of JAs biosynthesis with respect to wild type cells (Figure 5A). By contrast, mutant ∆*orf8-araC* showed a slight increase of **1** and **2** production, concomitant with a diminished JA A level. As a second assessment of the potential involvement of these genes in JAs biosynthesis, they were ectopically expressed under the control of the constitutive promoter ermE*p from the integrative plasmid pSETk. The resulting constructs pSAraC, pSLuxR, pSMarR and pSGntRwere were transferred to wild type *S. caniferus* GUA-06-05-006A to produce the recombinant strains G-pSAraC, G-pSLuxR, G-pSMarR and G-pSGntR, respectively, which were likewise subjected to metabolite production analysis (Figure 5B). Overexpression of *orf29* (G-pSGntR) resulted in JAs biosynthesis abrogation and a severe impairment of PM100117/18 production, indicating the pleiotropic effect possibly exerted by this gene on metabolite production. This observation, along with the unaltered biosynthesis of compounds **1** and **2** upon *orf29* deletion, makes us to consider that this *orf* lies outside the JAs biosynthesis gene cluster. Pleiotropic effects on PM100117/18 biosynthesis are similarly observed in recombinant strains G-pSLuxR (*orf16*) and G-pSMarR (*orf20*), but neither these strains nor G-pSAraC (*orf8*) showed altered **1** and **2** biosynthesis levels relative to control. Under the light of these observations, it is not possible to determine whether *orf8* and *orf29* play a direct role or simply exert a pleiotropic effect on JAs biosynthesis.

The right boundary of the JAs gene cluster is thus defined by *jomM*, as the adjacent *orf16* does not seem to be involved in JAs biosynthesis. This side of the cluster also comprises putative gene functions connected to chemical detoxification (*orf21* and *orf30*), DNA cleavage and repair (*orf19* and *orf22*) and primary metabolism processes, such as sugar metabolism and transport (*orf24*-*orf28*) and nitrogen metabolism (*orf31* and *orf32*). The left limit of the cluster might be defined by *jomP1*, whose adjacent *orf14* and *orf15* encode for hypothetical and unassigned function proteins, respectively. Besides, this flank of the JAs gene cluster contains miscellanea of hypothetical gene functions related to virulence (*orf4*, *orf7* and *orf11*), cell division (*orf3*) and cell signaling (*orf10*) among others (Table 1).

### 2.4. Heterologous Expression of the JAs BGC

**Figure 6:**
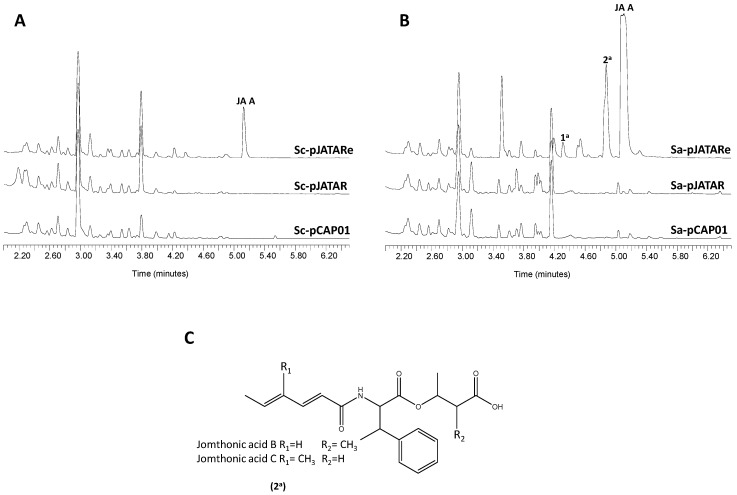
Heterologous expression of the jomthonic acids biosynthesis gene cluster. UPLC metabolite analysis of (**A**) *Streptomyces coelicolor* M1152 and (**B**) *Streptomyces albus* J1074 carrying plasmid pCAP01 (empty plasmid), pJATAR (JAs BGC) and pJATARe (JAs BGC with ermE*pinserted). (**C**) Speculative chemical structure of peaks 2^a^ (jomthonic acids B or C) in *S. albus* J1074.

In order to confirm the involvement of the proposed gene cluster in JA biosynthesis, a DNA fragment spanning from *orf9* to *orf24* was cloned using the transformation-associated recombination (TAR) cloning system [37] and the resulting construct, pJATAR, was transferred to the heterologous hosts *Streptomyces coelicolor* M1152 and *Streptomyces albus* J1074 to produce strains Sc-pJATAR and Sa-pJATAR, respectively. These strains were fermented in a culture medium that favors secondary metabolite biosynthesis in their respective backgrounds. Analysis by UPLC of organic extracts from the resulting cultures did not detect traces of JAs (Figure 6). This result led us to hesitate on the expression in the heterologous hosts of genes presumably required for JAs biosynthesis. Thus, we manipulated pJATAR to insert the constitutive promoter ermE*p in front of *jomP1* to compel transcription of genes *jomP1*-*jomM*, which possibly constitutes a single transcriptional unit. Likewise, the resulting plasmid, pJATARe, was introduced in *S. coelicolor* M1152 and *S. albus* J1074, generating strains Sc-pJATARe and Sa-pJATARe, respectively. Following UPLC analysis of metabolite production the presence of JA A biosynthesis was detected in both recombinant strains (Figure 6). Besides, based on absorption spectra resemblance, strain Sa-pNTARe produced two additional potential JAs family members, peaks 1^a^ and 2^a^ (Figure 6B). Mass spectra of these products are shown in Supplementary file 2: Figure S24. Molecular weight of product 2^a^ matched that of JA B/C (*m*/*z*374 [*M*+H]^+^) (Figure 6C), which remained undetected in the natural producer *S. caniferus* GUA-06-05-006A (Figure 2). Later metabolite analysis of 5-fold concentrated culture extracts from the *S. caniferus* GUA-06-05-006A derivative strain G-permE* led to the detection, by absorption spectra resemblance, of other potential JA candidates, including JA F (Supplementary file 2: Figure S25), but not JA B/C. Given their low production levels, purification of these compounds did not yield the product quantities required to carry out NMR analysis.

## 3. Discussion

Given their adipogenic activity, JAs constitute a promising family of NPs in metabolic disorders research [57,58,59]. JA’s chemical structure contains interesting structural moieties also reported in other metabolites. For instance, the JA A short polyketide chain is identical to that of salinamide C from *Streptomyces* sp. CNB-091 [60] and daldinin F from *Hypoxylonfuscum* [61]. Likewise, βMePhe is an unusual non-proteinogenic amino acid previously described as part of the altemicidin derivative SB-203208 [62], bottromycins [63], AK toxin [64], homaomycin [65] and mannopeptimycin [66]. However, only the latter two compounds share with JAs the 3R configuration of the βMePhe stereocenter.

Following a molecular elicitation strategy, we have identified the JA BGC, which has been characterized based on bioinformatics analysis and genetic engineering data. The proposed cluster covers 17.1 Kb and contains six biosynthesis genes comprised between *jomP1* and *jomM*, coding for putative functions required for polyketide biosynthesis and phenylalanine methylation. PKS JomP1 displays an uncommon structure, consisting of two ACP domains and two adjacent AT domains, up to date only reported in members of myxobacteria [46,47,48,49]. For similar myxobacterial PKS genes, a mechanism by which the first AT domain transfers an unconventional starting unit and the second AT domain transfer either malonyl- or methylmalonyl-CoA has been proposed. However, if JomP1 operated through this mechanism, it would be arduous to assess the type of starting and elongation units employed to synthesize the short polyketide chain present in JAs. Tentatively, we could postulate malonyl- and methylmalonyl-CoA as starting and extension molecules, respectively, which along with latter condensation to malonyl-CoA catalyzed by JomP2 could generate the JA polyketide chain with an extra hydroxyl group at C2 (Supplementary file 2: Figure S26). Instead, based on the sequence of conserved domain, we have considered plausible the inactivity of AT_2_ and transfer of MEB-CoA by AT_1_ domain (Figure 3).

Despite the absence of information on various gene functions presumably required for JA biosynthesis, we have intuitively outlined a biosynthesis pathway (Figure 3). Although no traces of JA B and C have been detected in *S. caniferus* GUA-06-05-006A, biosynthesis of both compounds could occur through similar reaction steps as JA A when 2-eno-butyrate and 3-hydroxybutyrate are available as substrates for JomP1 and enzyme X_1_, respectively (Figure 3). Based on predicted gene functions, enzymes relevant to MHB transfer seem to be absent from the predicted JAs BGC. Thus, according to the enzymatic activities encoded within the proposed biosynthesis pathway, it could be reasonable to consider JA F as the true pathway end product, and the other JAs family members as pathway shunt metabolites. Interestingly, heterologous expression of the BGC led to the biosynthesis of an array of JAs in *S. albus* J1074, including JA A and possibly JA B or C. Whether the enzymatic activity required for MHB transfer to JA F has been provided in the heterologous DNA fragment (unlikely) or, on the contrary, is integrated in the host genome is unknown. Detection of additional JA derivatives in a heterologous host reinforces the idea that the biosynthetic machinery harbored in different actinobacteria lineages can be successfully combined and exploited to generate structural diversity.

Expression of the JA BGC seems to be regulated, as no JAs are detected in the heterologous host unless its biosynthesis is elicited by ermE*p insertion upstream of *jomP1*. Gene deletion and overexpression experiments have not demonstrated the direct involvement of putative transcriptional regulators coded in the surroundings of *jomP1*-*jomM* on JAs biosynthesis control. Hence, based on these results, presence of key regulators encoded outside the JAs BGC is foreseeable. Lack of essential gene functions in BGC is frequently explained on the basis of large-scale genetic rearrangements, such as inversions and transpositions, which are fundamental to genome evolution [67,68].

This work provides a first insight into the genetic bases of JAs biosynthesis and shows heterologous expression of BGCs as a suitable strategy to generate additional structural diversity. Moreover, availability of the JAs BGC enables further genetic manipulations aimed at generating novel derivatives with improved pharmacological properties and eases the elucidation of unraveled biosynthesis steps.

## 4. Materials and Methods

### 4.1. Strains, Tumor Cell Lines, Media and Cultivation Conditions

*Streptomyces* strains *S. caniferus* GUA-06-05-006A [69], *S. coelicolor* M1152 [70] and *S. albus* J1074 [36] were routinely maintained in medium A (MA) [71]. Metabolite production analyses and large scale fermentations in *S.caniferus* GUA-06-05-006A were performed in SMS medium as described elsewhere [44]. Heterologous production of JAs in *S. coelicolor* M1152 and *S. albus* J1074 was achieved after 3 days of cultivation at 30 °C in GYM [72] and R5A medium [71], respectively. *Escherichia coli* strains DH10B [73], used for cloning, ET12567/pUB307 [74] and ET12567/pUZ8002 [75], used for intergeneric conjugation, were grown in LB or 2× TY medium, supplemented with the appropriate antibiotic for plasmid selection [74].

Yeast strain *Saccharomyces cerevisiae* VL6-48 (MAT alpha, his3-D200, trp1-D1, ura3-52, lys2, ade2-101, met14, psi + cir0), used for TAR cloning, was grown in YPD or YNB-trp medium [76], made solid, when required, by addition of 2% agar.

### 4.2. DNA Manipulation

DNA manipulation in *Escherichia coli* and *S. caniferus* GUA-06-05-006A was carried out according to standard protocols [74,77]. Polymerase chain reaction (PCR) amplifications were performed by using Herculase II Fusion polymerase (Agilent Technologies, Palo Alto, CA, USA) with a previously reported touchdown PCR procedure [44]. All primers used in this work are described in Supplementary file 3: Table S1. Genetic manipulations in *S. caniferus* GUA-06-05-006A were confirmed by colony PCR by using the procedure described in [78] with the following modifications. Bacterial colonies were suspended in 50 μL of 0.2 M 2-[[1,3-dihydroxy-2-(hydroxymethyl)propan-2-yl]amino] ethanesulfonic acid (TES) buffer, pH 7.5, with 1 μL lysozyme (50 mg/mL) and incubated for 40 min at 30 °C. The mix was centrifuged (10,000× *g*, 2 min) and the pellet thoroughly suspended in 10 μL dimethyl sulfoxide (DMSO). The resulting suspension (2 μL) was used as PCR template with the indicated primer pairs (Supplementary file 3: Table S1).

### 4.3. Plasmids Construction and Strain Generation

Information relevant to plasmids generated in this work is summarized in Supplementary file 3: Table S1. All plasmids were transferred to *S.caniferus* GUA-06-05-006Aby intergeneric conjugation as previously described [44]. For *jomP1* and *jomN* insertional inactivation, internal gene fragments were amplified with the primer pairs EcoRI-P1/ HindIII-P1 and EcoRI-N/ HindIII-N, respectively, and cloned in the indicated restriction sites of plasmid pOJ260 [79], which lacks the capacity to replicate in *Streptomyces* and carries the *aac*(*3*)*IV* gene marker that confers resistance to apramycin(Am^R^). The resulting plasmids, pIjomP1 and pIjomN, were then transferred to *S.caniferus* GUA-06-05-006A to produce strains ∆*jomP1* and ∆*jomN*, respectively. Insertion of ermE*p upstream of *jomP1* was performed in the pOJ260 derivative pOJ260e as described in [36]. A 2.8 Kb fragment covering the *jomP1* upstream region, including 108 bp from the start codon, was amplified with primers EcoRI-EP1/PstI-EP1 and cloned in pOJ260e to afford plasmid pEjomP1, which was used to generate strain G-permE*.

To accomplish single deletion of *orf8*, *orf9*, *orf16*, *orf20*, *orf29*, *orf31* and *jomM*, the downstream DNA sequence of the referred target genes were amplified with the primer pairsBamHI-orf8/EcoRV-orf8, BglIIHI-orf9/EcoRV-orf9, BamHI-orf16/EcoRV-orf16, BamHI-orf20/EcoRV-orf20, BglIIHI-orf29/EcoRV-orf29, BglIIHI-orf31/EcoRV-orf31 and BglIIHI-M/EcoRV-M, respectively, and cloned in the designated restriction sites of plasmid pEFBA-oriT [80], downstream to the *aac*(*3*)*IV* gene. Then, the upstream sequence of the cited genes were likewise amplified with the primer pairs NsiI-orf8/SpeI-orf8, NsiI-orf9/SpeI-orf9, NsiI-orf16/SpeI-orf16, NsiI-orf20/SpeI-orf20, NsiI-orf29/SpeI-orf29, NsiI-orf31/SpeI-orf31 andNsiI-M/SpeI-M, respectively, and cloned upstream to the *aac*(*3*)*IV* gene. Finally, the hygromycin B resistance (Hyg^R^) gene marker, *hyg*, was extracted by XbaI/NheI digestion from plasmid pLHyg [81] and introduced in the XbaI site of the deletion plasmids. The resulting construction plasmids, pDorf8, pDorf9, pDorf16, pDorf20, pDorf29, pDorf31, pDjomM were introduced in *S.caniferus* GUA-06-05-006A to generate deletion strains ∆orf8-araC, ∆*orf9*, ∆*orf16-luxR*, ∆*orf20-marR*, ∆*orf29-gntR*, ∆*orf31* and ∆*jomM*, respectively. Gene *hyg* allowed recognizing clones in which a complete gene replacement by a double cross-over has taken place (Hyg^S^ Am^R^) from those in which a single cross-over event has integrated the deletion plasmid into the chromosome (Hyg^R^ Am^R^).

A suitable plasmid backbone to accomplish ectopic expression was constructed as follows. A 930 bp fragment containing the kanamycin resistance marker *aph*(*3*) *II* was amplified from plasmid pCAP01 [37] with primers EcoRI-KanR/EcoRV-KanR and cloned in the integrative plasmid pSETec [82], contiguous to the constitutive ermE*p promoter to produce plasmid pSETk. Genes *orf8*, *orf16*, *orf20*, *orf29* and *jomM* were amplified with primers indicated in Supplementary file 3: Table S1, and cloned in pSETk between ermE*p and *aph(3) II*. The resulting plasmids (Supplementary file 3: Table S1) were transferred to *S.caniferus* GUA-06-05-006A or ∆jomMto generate strains G-pSAraC, G-pSLuxR, G-pSMarR, G-pSGntR and ∆M-pSEJomM.

### 4.4. Heterologous Expression of the JAs BGC

The JAs BGC (*orf9* to *orf23*) was captured by TAR cloning [37] for heterologous expression in *S. coelicolor* M1152 and *S. albus* J1074. Cluster capture arms of approximately 1 Kb were generated by PCR amplification with primer pairs XhoI-2TAR/NsiI-U2TAR and NsiI-L2TAR/SpeI-2TAR (Supplementary file 3: Table S1). PCR products were digested with NsiI and ligated. The resulting assembled fragment of 2 Kb was then amplified with primers XhoI-2TAR/SpeI-2TAR and cloned in the XhoI/SpeI sites of plasmid pCAP01 to afford the capture plasmid pCL2-CAP. Yeast strain *Saccharomyces cerevisiae* VL6-48 was then co-transformed [76] with pCL2-CAP (0.5 μg), linearized by digestion with NsiI, and *S. caniferus*GUA-06-05-006A genomic DNA (1 μg). Yeast transformants were selected on synthetic YNB–trp medium and screened by PCR for the presence of the JAs BGC. The resulting plasmid, p2NTAR, was extracted from three positive clones, subjected to physical characterization by BamHI, NcoI, NotI, PstI and EcoRV digestion and transferred to the heterologous *Streptomyces* hosts by intergeneric conjugation [44].

To produce plasmid pNTARe, the ermE*p promoter was integrated upstream of *jomP1*, in plasmid pJATAR, by ReDirect technology [83].The apramycin resistant gene marker *aac*(*3*)*IV* and ermE*p were amplified from plasmid pSETec [82] with the primer pairs dApra/NsiI-rvApra and dNsiI-pErm/rvpErm, respectively. The PCR products were digested with NsiI and ligated. The resulting *aac*(*3*)*IV*-ermE*p assembled fragment was then amplified with the primer pair dApra-pErm/rvApra-pErm to generate a DNA fragment flanked with suitable pJATAR homologous sequences to direct ermE*p integration upstream of *jomP1*. This fragment and pJATAR were introduced in *E. coli* BW25113 as described in [83]. Kanamycin/apramycin resistant transformants were selected and analyzed for correct ermE*p integration by sequencing with primers dNsiI-pErm, rvpErm, dApra and NsiI-rvApra.

### 4.5. Sequencing and Bioinformatics Analysis

The JAs BGC was identified and analyzed by the antibiotics and secondary metabolite analysis shell: antiSMASH 4.0 [84] and PRISM 3 [85,86]. Annotation of ORFs within the JA BGC was based on database searching of the corresponding proteins carried out by BLAST algorithm [87] at the National Center for Biotechnology Information (NCBI). Additional sequence alignments were conducted by Clustal Omega [88] and EMBOSS Water [89] from the European Molecular Biology Laboratory (EMBL).

The nucleotide sequence of the jomthonic acids biosynthesis gene cluster was deposited in the European Nucleotide Archive (accession number: LT990689) and at Minimum Information about a Biosynthetic Gene Cluster (MIBiG) repository [90] under the accession BGC0001457.

### 4.6. Analysis of Metabolite Production and Compound Purification

Whole culture samples (1 or 5 mL) were mixed with an equal volume of ethyl acetate and mixed on a rotary shaker at room temperature for 1 h. The organic phase was then recovered by centrifugation (3000× *g*, 10 min) and evaporated in vacuo. The resulting dry residue was dissolved in methanol:DMSO (1:1) to perform UPLC and LC-MS analyses as described elsewhere [91,92].

Compounds **1**–**3** were purified from 2.5 L of G-permE* culture supernatant. Culture was filtered and then applied to a solid-phase extraction cartridge (Sep-Pak Vac C18, 10 g, Waters, Mildford, MA, USA) that had been fitted with a perforated stopper pierced by a stainless steel HPLC (Waters, Mildford, MA, USA) tubing. The culture broth was applied by means of a peristaltic pump and subsequently the cartridge was connected to a HPLC quaternary pump (model 600E, Waters). The retained material was eluted with a mixture of methanol and 0.05% trifluoroacetic acid (TFA) in water. A linear gradient from 0 to 100% methanol in 60 min, at 10 mL/min, was used. Fractions were taken every 5 min, collected on 5 mL of 0.1 M phosphate buffer, pH 7.0 and analyzed by UPLC. Fractions containing the desired compounds were evaporated in vacuo and subsequently re-dissolved in a small volume of a DMSO:methanol (50:50) mixture. Compounds of interest were then purified by preparative HPLC using a SunFire C18 column (10 µm, 10 × 250 mm, Waters, Mildford, MA, USA). Organic extracts were chromatographed with mixtures of acetonitrile or methanol and 0.1% TFA in accurate isocratic conditions (7 mL/min), and collected on 0.1 M phosphate buffer (pH 7.0). After every purification step, the collected compounds were diluted 4-fold with water and then applied to a solid-phase extraction cartridge (Sep-Pak C18, Waters, Mildford, MA, USA). The cartridges were washed with water and compounds were eluted with methanol and dried in vacuo. Once the purification was finished, the compounds were dissolved in a mixture of tert-butanol and water (1:1) and lyophilized, obtaining 21.9, 3.8 and 3.9 mg of compounds 1, 2 and 3, respectively.

### 4.7. Mass Spectra and Structural Elucidation

NMR spectra were obtained on a Varian “Unity 500” spectrometer (Agilent Technologies, Palo Alto, CA, USA) at 500/125 MHz (1H/13C) and on a Varian “Unity 400” spectrometer (Agilent Technologies, Palo Alto, CA, USA) at 400/100 MHz (1H/13C). Chemical shifts were reported in ppm using residual CDCl3 (δ 7.26 ppm for 1H and 77.0 ppm for 13C) as an internal reference. COSY, HSQC and HMBC experiments were performed using standard pulse sequences. Data were processed using MestReNova software (Mnova, Santiago de Compostela, Spain). (+)ESIMS were recorded using an Agilent 1100 Series LC/MS spectrometer (Agilent Technologies, Palo Alto, CA, USA). High resolution mass spectroscopy (HRMS) was performed on an Agilent 6230 TOF LC/MS system (Agilent Technologies, Palo Alto, CA, USA) using the electrospray ionization mass spectrometry (ESI-MS) technique.

## Figures and Tables

**Table 1:** Deduced functions of open reading frames (ORFs) in jomthonic acids biosynthetic gene cluster.

Gene	Deduced aa. Length	Protein Homologue (Accession No.)	Identity/Similarity (%)	Proposed Function
*orf1*	194	WP_030077520.1	93/86	RNA polymerase
*orf2*	313	WP_053208602.1	95/96	Hypothetical protein
*orf3*	1462	WP_084771961.1	94/96	Cell division protein FtsK
*orf4*	1525	WP_055485092.1	59/70	Type IV secretion protein Rhs
*orf5*	241	WP_084771964.1	93/95	Hypothetical protein
*orf6*	104	WP_084771965.1	86/94	Hypothetical protein
*orf7*	99	WP_033268020.1	99/100	WXG-like protein
*orf8*	289	SDQ83237.1	89/91	AraC-family transcriptional regulator
*orf9*	396	SDQ83203.1	87/93	Monooxygenase
*orf10*	315	OSY47464.1	92/94	FG-GAP repeat protein
*orf11*	95	OSY47461.1	96/96	Type II toxin–antitoxin system
*orf12*	286	OSY47460.1	91/94	Thioesterase
*orf13*	218	WP_085923163.1	99/99	ATP-dependent Clp protease proteolytic subunit
*orf14*	106	OSY47458.1	95/97	Hypothetical protein
*orf15*		-	-	Unassigned
*jomP1*	2093	WP_020735335.1	47/58	Type I polyketide synthase (PCP/KS/AT/AT/KR/PCP)
*jomP2*	1853	CAD19087.1	43/57	Type I polyketide synthase (KS/AT/DH/KR/PCP)
*jomN*	1062	SCL52665.1	48/63	Non-ribosomal protein synthetase(C/A/PCP)
*jomT*	254	WP_025675064.1	35/51	Thioesterase
*jomB*	72	WP_004933768.1	72/83	*mbt*H-like protein
*jomM*	329	WP_004933770.1	78/85	Methyltransferase
*orf16*	751	WP_053684324.1	53/65	LuxR-family transcriptional regulator
*orf17*	229	WP_042159102.1	83/88	Hypothetical protein
*orf18*	404	WP_052718894.1	90/93	Hypothetical protein
*orf19*	295	SCK58100.1	86/91	Putative nuclease
*orf20*	170	WP_035838695.1	81/85	MarR-family transcriptional regulator
*orf21*	123	WP_039639044.1	93/95	Glyoxalase
*orf22*	318	WP_042157776.1	78/83	DNA-3-methyladenine glycosylase 2 family protein
*rf23*	190	WP_042159103.1	97/99	Hypotheticalprotein
*orf24*	373	WP_042159104.1	99/100	ROK-familyglucokinase
*orf25*	269	WP_085927837.1	100/100	Sugar ABC transporter ATP-binding protein
*orf26*	356	WP_085927838.1	99/99	Ribose ABC transporter permease
*orf27*	337	WP_042159107.1	99/100	Sugar ABC transporter substrate-binding protein
*orf28*	358	WP_042159108.1	96/97	Sugar ABC transporter substrate-binding protein
*orf29*	258	GAO11207.1	98/99	GntR-family transcriptional regulator
*orf30*	126	WP_018092820.1	84/90	Glyoxalase
*orf31*	275	WP_042159111.1	95/96	Putative aminoacid ABC transporter
*orf32*	219	WP_085927844.1	99/99	2-dehydro-3-deoxy-phosphogluconate aldolase

## Supplementary Materials

The following are available online at http://www.mdpi.com/1660-3397/16/8/259/s1, Supplementary file 1: Figure S1–S21, Supplementary file 2: Figure S22–S26, Supplementary file 3: Table S1 and Figure S27.

## References

[1] Waters A.L., Hill R.T., Place A.R., Hamann M.T. (2010). The expanding role of marine microbes in pharmaceutical development. Curr. Opin. Biotechnol..

[2] Snelgrove P.V.R. (2016). An Ocean of Discovery: Biodiversity Beyond the Census of Marine Life. Planta Med..

[3] Williams J.A., Day M., Heavner J.E. (2008). Ziconotide: An update and review. Expert Opin. Pharmacother..

[4] Yasuhara-Bell J., Lu Y. (2010). Marine compounds and their antiviral activities. Antiviral Res..

[5] Cuevas C., Francesch A. (2009). Development of Yondelis (trabectedin, ET-743). A semisynthetic process solves the supply problem. Nat. Prod. Rep..

[6] Stonik V.A. (2009). Marine natural products: a way to new drugs. Acta Nat..

[7] Anjum K., Abbas S.Q., Akhter N., Shagufta B.I., Shah S.A.A., Hassan S.S.U. (2017). Emerging biopharmaceuticals from bioactive peptides derived from marine organisms. Chem. Biol. Drug Des..

[8] Sipkema D., Franssen M.C.R., Osinga R., Tramper J., Wijffels R.H. (2005). Marine sponges as pharmacy. Mar. Biotechnol. (N.Y.).

[9] Anjum K., Abbas S.Q., Shah S.A.A., Akhter N., Batool S., Hassan S.S. (2016). ul Marine Sponges as a Drug Treasure. Biomol. Ther. (Seoul).

[10] Bernan V.S., Greenstein M., Maiese W.M. (1997). Marine microorganisms as a source of new natural products. Adv. Appl. Microbiol..

[11] Simmons T.L., Coates R.C., Clark B.R., Engene N., Gonzalez D., Esquenazi E., Dorrestein P.C., Gerwick W.H. (2008). Biosynthetic origin of natural products isolated from marine microorganism-invertebrate assemblages. Proc. Natl. Acad. Sci. USA.

[12] Romano G., Costantini M., Sansone C., Lauritano C., Ruocco N., Ianora A. (2017). Marine microorganisms as a promising and sustainable source of bioactive molecules. Mar. Environ. Res..

[13] Sarmiento-Vizcaíno A., González V., Braña A.F., Palacios J.J., Otero L., Fernández J., Molina A., Kulik A., Vázquez F., Acuña J.L. (2017). Pharmacological Potential of Phylogenetically Diverse Actinobacteria Isolated from Deep-Sea Coral Ecosystems of the Submarine Avilés Canyon in the Cantabrian Sea. Microb. Ecol..

[14] Brady S.F., Simmons L., Kim J.H., Schmidt E.W. (2009). Metagenomic approaches to natural products from free-living and symbiotic organisms. Nat. Prod. Rep..

[15] Heidelberg K.B., Gilbert J.A., Joint I. (2010). Marine genomics: At the interface of marine microbial ecology and biodiscovery. Microb. Biotechnol..

[16] Mühling M., Joint I., Willetts A.J. (2013). The biodiscovery potential of marine bacteria: An investigation of phylogeny and function. Microb. Biotechnol..

[17] Amoutzias G., Chaliotis A., Mossialos D. (2016). Discovery Strategies of Bioactive Compounds Synthesized by Nonribosomal Peptide Synthetases and Type-I Polyketide Synthases Derived from Marine Microbiomes. Mar. Drugs.

[18] Singh M., Chaudhary S., Sareen D. (2017). Non-ribosomal peptide synthetases: Identifying the cryptic gene clusters and decoding the natural product. J. Biosci..

[19] Tseng C.-H., Tang S.-L. (2014). Marine Microbial Metagenomics: From Individual to the Environment. Int. J. Mol. Sci..

[20] Uchiyama I., Mihara M., Nishide H., Chiba H. (2015). MBGD update 2015: Microbial genome database for flexible ortholog analysis utilizing a diverse set of genomic data. Nucleic Acids Res..

[21] Haroon M.F., Thompson L.R., Parks D.H., Hugenholtz P., Stingl U. (2016). A catalogue of 136 microbial draft genomes from Red Sea metagenomes. Sci. Data.

[22] Katz L., Baltz R.H. (2016). Natural product discovery: Past, present, and future. J. Ind. Microbiol. Biotechnol..

[23] Jensen P.R. (2016). Natural Products and the Gene Cluster Revolution. Trends Microbiol..

[24] Niu G. (2018). Genomics-Driven Natural Product Discovery in Actinomycetes. Trends Biotechnol..

[25] Challis G.L. (2008). Mining microbial genomes for new natural products and biosynthetic pathways. Microbiology.

[26] McAlpine J.B. (2009). Advances in the understanding and use of the genomic base of microbial secondary metabolite biosynthesis for the discovery of new natural products. J. Nat. Prod..

[27] Nett M., Ikeda H., Moore B.S. (2009). Genomic basis for natural product biosynthetic diversity in the actinomycetes. Nat. Prod. Rep..

[28] Li J.W.-H., Vederas J.C. (2009). Drug Discovery and Natural Products: End of an Era or an Endless Frontier?. Science.

[29] Abdelmohsen U.R., Grkovic T., Balasubramanian S., Kamel M.S., Quinn R.J., Hentschel U. (2015). Elicitation of secondary metabolism in actinomycetes. Biotechnol. Adv..

[30] Reen F.J., Romano S., Dobson A.D.W., O’Gara F. (2015). The Sound of Silence: Activating Silent Biosynthetic Gene Clusters in Marine Microorganisms. Mar. Drugs.

[31] Baltz R.H. (2016). Genetic manipulation of secondary metabolite biosynthesis for improved production in Streptomyces and other actinomycetes. J. Ind. Microbiol. Biotechnol..

[32] Ochi K. (2017). Insights into microbial cryptic gene activation and strain improvement: Principle, application and technical aspects. J. Antibiot. (Tokyo).

[33] Jensen P.R., Chavarria K.L., Fenical W., Moore B.S., Ziemert N. (2014). Challenges and triumphs to genomics-based natural product discovery. J. Ind. Microbiol. Biotechnol..

[34] Gomez-Escribano J., Alt S., Bibb M. (2016). Next Generation Sequencing of Actinobacteria for the Discovery of Novel Natural Products. Mar. Drugs.

[35] Luo Y., Huang H., Liang J., Wang M., Lu L., Shao Z., Cobb R.E., Zhao H. (2013). Activation and characterization of a cryptic polycyclic tetramate macrolactam biosynthetic gene cluster. Nat. Commun..

[36] Olano C., García I., González A., Rodriguez M., Rozas D., Rubio J., Sánchez-Hidalgo M., Braña A.F., Méndez C., Salas J.A. (2014). Activation and identification of five clusters for secondary metabolites in Streptomyces albus J1074. Microb. Biotechnol..

[37] Yamanaka K., Reynolds K.A., Kersten R.D., Ryan K.S., Gonzalez D.J., Nizet V., Dorrestein P.C., Moore B.S. (2014). Direct cloning and refactoring of a silent lipopeptide biosynthetic gene cluster yields the antibiotic taromycin A. Proc. Natl. Acad. Sci. USA.

[38] Saha S., Zhang W., Zhang G., Zhu Y., Chen Y., Liu W., Yuan C., Zhang Q., Zhang H., Zhang L. (2017). Activation and characterization of a cryptic gene cluster reveals a cyclization cascade for polycyclic tetramate macrolactams. Chem. Sci..

[39] Frattaruolo L., Lacret R., Cappello A.R., Truman A.W. (2017). A Genomics-Based Approach Identifies a Thioviridamide-Like Compound with Selective Anticancer Activity. ACS Chem. Biol..

[40] Xu J., Zhang J., Zhuo J., Li Y., Tian Y., Tan H. (2017). Activation and mechanism of a cryptic oviedomycin gene cluster via the disruption of a global regulatory gene, adpA, in Streptomyces ansochromogenes. J. Biol. Chem..

[41] Kawahara T., Izumikawa M., Kozone I., Hashimoto J., Kagaya N., Koiwai H., Komatsu M., Fujie M., Sato N., Ikeda H. (2018). Neothioviridamide, a Polythioamide Compound Produced by Heterologous Expression of a Streptomyces sp. Cryptic RiPP Biosynthetic Gene Cluster. J. Nat. Prod..

[42] Igarashi Y., Yu L., Ikeda M., Oikawa T., Kitani S., Nihira T., Bayanmunkh B., Panbangred W. (2012). Jomthonic acid A, a modified amino acid from a soil-derived Streptomyces. J. Nat. Prod..

[43] Yu L., Oikawa T., Kitani S., Nihira T., Bayanmunkh B., Panbangred W., Igarashi Y. (2014). Jomthonic acids B and C, two new modified amino acids from Streptomyces sp.. J. Antibiot. (Tokyo).

[44] Salcedo R.G., Olano C., Gómez C., Fernández R., Braña A.F., Méndez C., de la Calle F., Salas J.A. (2016). Characterization and engineering of the biosynthesis gene cluster for antitumor macrolides PM100117 and PM100118 from a marine actinobacteria: Generation of a novel improved derivative. Microb. Cell Fact..

[45] Magarvey N.A., Haltli B., He M., Greenstein M., Hucul J.A. (2006). Biosynthetic pathway for mannopeptimycins, lipoglycopeptide antibiotics active against drug-resistant gram-positive pathogens. Antimicrob. Agents Chemother..

[46] Gaitatzis N., Silakowski B., Kunze B., Nordsiek G., Blöcker H., Höfle G., Müller R. (2002). The biosynthesis of the aromatic myxobacterial electron transport inhibitor stigmatellin is directed by a novel type of modular polyketide synthase. J. Biol. Chem..

[47] Silakowski B., Schairer H.U., Ehret H., Kunze B., Weinig S., Nordsiek G., Brandt P., Blöcker H., Höfle G., Beyer S. (1999). New lessons for combinatorial biosynthesis from myxobacteria. The myxothiazol biosynthetic gene cluster of Stigmatella aurantiaca DW4/3-1. J. Biol. Chem..

[48] Silakowski B., Nordsiek G., Kunze B., Blöcker H., Müller R. (2001). Novel features in a combined polyketide synthase/non-ribosomal peptide synthetase: The myxalamid biosynthetic gene cluster of the myxobacterium Stigmatella aurantiaca Sga15. Chem. Biol..

[49] Ligon J., Hill S., Beck J., Zirkle R., Molnár I., Zawodny J., Money S., Schupp T. (2002). Characterization of the biosynthetic gene cluster for the antifungal polyketide soraphen A from Sorangium cellulosum So ce26. Gene.

[50] Petković H., Sandmann A., Challis I.R., Hecht H.-J., Silakowski B., Low L., Beeston N., Kuščer E., Garcia-Bernardo J., Leadlay P.F. (2008). Substrate specificity of the acyl transferase domains of EpoC from the epothilone polyketide synthase. Org. Biomol. Chem..

[51] Liew C.W., Nilsson M., Chen M.W., Sun H., Cornvik T., Liang Z.-X., Lescar J. (2012). Crystal structure of the acyltransferase domain of the iterative polyketide synthase in enediyne biosynthesis. J. Biol. Chem..

[52] Huang Y.-T., Lyu S.-Y., Chuang P.-H., Hsu N.-S., Li Y.-S., Chan H.-C., Huang C.-J., Liu Y.-C., Wu C.-J., Yang W.-B. (2009). In vitro characterization of enzymes involved in the synthesis of nonproteinogenic residue (2S,3S)-beta-methylphenylalanine in glycopeptide antibiotic mannopeptimycin. ChemBiochem.

[53] Wu W.M., Jain M.K., Zeikus J.G. (1994). Anaerobic degradation of normal- and branched-chain Fatty acids with four or more carbons to methane by a syntrophic methanogenic triculture. Appl. Environ. Microbiol..

[54] Narihiro T., Nobu M.K., Tamaki H., Kamagata Y., Sekiguchi Y., Liu W.-T. (2016). Comparative Genomics of Syntrophic Branched-Chain Fatty Acid Degrading Bacteria. Microbes Environ..

[55] Felnagle E.A., Barkei J.J., Park H., Podevels A.M., McMahon M.D., Drott D.W., Thomas M.G. (2010). MbtH-like proteins as integral components of bacterial nonribosomal peptide synthetases. Biochemistry.

[56] Zolova O.E., Garneau-Tsodikova S. (2012). Importance of the MbtH-like protein TioT for production and activation of the thiocoraline adenylation domain of TioK. Medchemcomm.

[57] Camp H.S., Ren D., Leff T. (2002). Adipogenesis and fat-cell function in obesity and diabetes. Trends Mol. Med..

[58] Kassotis C.D., Masse L., Kim S., Schlezinger J.J., Webster T.F., Stapleton H.M. (2017). Characterization of Adipogenic Chemicals in Three Different Cell Culture Systems: Implications for Reproducibility Based on Cell Source and Handling. Sci. Rep..

[59] Calabro P., Yeh E.T. (2007). Obesity, inflammation, and vascular disease: The role of the adipose tissue as an endocrine organ. Subcell. Biochem..

[60] Moore B.S., Trischman J.A., Seng D., Kho D., Jensen P.R., Fenical W. (1999). Salinamides, antiinflammatory depsipeptides from a marine streptomycete. J. Org. Chem..

[61] Quang D.N., Hashimoto T., Tanaka M., Stadler M., Asakawa Y. (2004). Cyclic azaphilones daldinins E and F from the ascomycete fungus Hypoxylon fuscum (Xylariaceae). Phytochemistry.

[62] Houge-Frydrych C.S., Gilpin M.L., Skett P.W., Tyler J.W. (2000). SB-203207 and SB-203208, two novel isoleucyl tRNA synthetase inhibitors from a Streptomyces sp. II. Structure determination. J. Antibiot. (Tokyo).

[63] Yamada T., Yagita M., Kobayashi Y., Sennari G., Shimamura H., Matsui H., Horimatsu Y., Hanaki H., Hirose T., Omura S. (2018). Synthesis and Evaluation of Antibacterial Activity of Bottromycins. J. Org. Chem..

[64] Takaoka S., Kurata M., Harimoto Y., Hatta R., Yamamoto M., Akimitsu K., Tsuge T. (2014). Complex regulation of secondary metabolism controlling pathogenicity in the phytopathogenic fungus Alternaria alternata. New Phytol..

[65] Rössner E., Zeeck A., König W.A. (1990). Elucidation of the Structure of Hormaomycin. Angew. Chem. Int. Ed. Engl..

[66] Fuse S., Koinuma H., Kimbara A., Izumikawa M., Mifune Y., He H., Shin-ya K., Takahashi T., Doi T. (2014). Total synthesis and stereochemistry revision of mannopeptimycin aglycone. J. Am. Chem. Soc..

[67] Dandekar T., Snel B., Huynen M., Bork P. (1998). Conservation of gene order: A fingerprint of proteins that physically interact. Trends Biochem. Sci..

[68] Ochman H., Moran N.A. (2001). Genes lost and genes found: Evolution of bacterial pathogenesis and symbiosis. Science.

[69] Pérez M., Schleissner C., Fernández R., Rodríguez P., Reyes F., Zuñiga P., de la Calle F., Cuevas C. (2015). PM100117 and PM100118, new antitumor macrolides produced by a marine Streptomyces caniferus GUA-06-05-006A. J. Antibiot. (Tokyo).

[70] Gomez-Escribano J.P., Bibb M.J. (2011). Engineering Streptomyces coelicolor for heterologous expression of secondary metabolite gene clusters. Microb. Biotechnol..

[71] Fernández E., Weissbach U., Sánchez Reillo C., Braña A.F., Méndez C., Rohr J., Salas J.A. (1998). Identification of two genes from Streptomyces argillaceus encoding glycosyltransferases involved in transfer of a disaccharide during biosynthesis of the antitumor drug mithramycin. J. Bacteriol..

[72] Hu H., Zhang Q., Ochi K. (2002). Activation of antibiotic biosynthesis by specified mutations in the rpoB gene (encoding the RNA polymerase beta subunit) of Streptomyces lividans. J. Bacteriol..

[73] Grant S.G.N., Jessee J., Bloom F.R., Hanahan D. (1990). Differential plasmid rescue from transgenic mouse DNAs into Escherichia coli methylation-restriction mutants. Proc. Natl. Acad. Sci. USA.

[74] Kieser T., Bibb M.J., Buttner M.J., Chater K.F., Hopwood D. (2000). Practical Streptomyces Genetics.

[75] Strain C5 Paranthaman S., Dharmalingam K. (2003). Intergeneric conjugation in streptomyces peucetius and streptomyces sp. Strain C5: Chromosomal Integration and Expression of Recombinant Plasmids Carrying the chiC Gene. Appl. Environ. Microbiol..

[76] Gietz R.D. (2014). Yeast Transformation by the LiAc/SS Carrier DNA/PEG Method. Methods in Molecular Biology (Clifton, N.J.).

[77] Sambrook J., Russell D.W. (2001). Molecular Cloning: A. Laboratory Manual.

[78] Van Dessel W., Van Mellaert L., Geukens N., Anné J. (2003). Improved PCR-based method for the direct screening of Streptomyces transformants. J. Microbiol. Methods.

[79] Bierman M., Logan R., O’Brien K., Seno E.T., Rao R.N., Schoner B.E. (1992). Plasmid cloning vectors for the conjugal transfer of DNA from Escherichia coli to Streptomyces spp.. Gene.

[80] Horna D.H., Gómez C., Olano C., Palomino-Schätzlein M., Pineda-Lucena A., Carbajo R.J., Braña A.F., Méndez C., Salas J.A. (2011). Biosynthesis of the RNA polymerase inhibitor streptolydigin in Streptomyces lydicus: Tailoring modification of 3-methyl-aspartate. J. Bacteriol..

[81] Olano C., Moss S.J., Braña A.F., Sheridan R.M., Math V., Weston A.J., Méndez C., Leadlay P.F., Wilkinson B., Salas J.A. (2004). Biosynthesis of the angiogenesis inhibitor borrelidin by Streptomyces parvulus Tü4055: Insights into nitrile formation. Mol. Microbiol..

[82] Cano-Prieto C., García-Salcedo R., Sánchez-Hidalgo M., Braña A.F., Fiedler H.-P., Méndez C., Salas J.A., Olano C. (2015). Genome Mining of Streptomyces sp. Tü 6176: Characterization of the Nataxazole Biosynthesis Pathway. ChemBioChem.

[83] Gust B., Kieser T., Chater K. (2002). Redirect Technology: PCR Targeting System in Streptomyces Coelicolor.

[84] Blin K., Wolf T., Chevrette M.G., Lu X., Schwalen C.J., Kautsar S.A., Suarez Duran H.G., de Los Santos E.L.C., Kim H.U., Nave M. (2017). Antismash 4.0-improvements in chemistry prediction and gene cluster boundary identification. Nucleic Acids Res..

[85] Skinnider M.A., Johnston C.W., Edgar R.E., Dejong C.A., Merwin N.J., Rees P.N., Magarvey N.A. (2016). Genomic charting of ribosomally synthesized natural product chemical space facilitates targeted mining. Proc. Natl. Acad. Sci. USA.

[86] Skinnider M.A., Dejong C.A., Rees P.N., Johnston C.W., Li H., Webster A.L.H., Wyatt M.A., Magarvey N.A. (2015). Genomes to natural products PRediction Informatics for Secondary Metabolomes (PRISM). Nucleic Acids Res..

[87] Altschul S.F., Madden T.L., Schäffer A.A., Zhang J., Zhang Z., Miller W., Lipman D.J. (1997). Gapped BLAST and PSI-BLAST: A new generation of protein database search programs. Nucleic Acids Res..

[88] Sievers F., Higgins D.G. (2014). Clustal Omega, Accurate Alignment of Very Large Numbers of Sequences. Methods in Molecular Biology (Clifton, N.J.).

[89] Rice P., Longden I., Bleasby A. (2000). EMBOSS: The European Molecular Biology Open Software Suite. Trends Genet..

[90] Medema M.H., Kottmann R., Yilmaz P., Cummings M., Biggins J.B., Blin K., de Bruijn I., Chooi Y.H., Claesen J., Coates R.C. (2015). Minimum Information about a Biosynthetic Gene cluster. Nat. Chem. Biol..

[91] Losada A.A., Cano-Prieto C., García-Salcedo R., Braña A.F., Méndez C., Salas J.A., Olano C. (2017). Caboxamycin biosynthesis pathway and identification of novel benzoxazoles produced by cross-talk in Streptomyces sp. NTK 937. Microb. Biotechnol..

[92] Schleissner C., Cañedo L.M., Rodríguez P., Crespo C., Zúñiga P., Peñalver A., de la Calle F., Cuevas C. (2017). Bacterial Production of a Pederin Analogue by a Free-Living Marine Alphaproteobacterium. J. Nat. Prod..

