# Supplementary material for: Characterization of the Jomthonic Acids Biosynthesis Pathway and Isolation of Novel Analogues in Streptomyces caniferus GUA-06-05-006A
Source: Mar Drugs. 2018 Jul 31;16(8):259. doi: 10.3390/md16080259 (PMC6117699; doi:10.3390/md16080259)

Table of contents **Pages**

Figures S1-S5. 1D and 2D NMR spectra of **1** in $CDCl_3$ 2-6

Figures S6-S11. 1D and 2D NMR spectra of **2** in $CDCl_3$. 7-12

Figures S12-S18. 1D and 2D NMR spectra of **3** in $CDCl_3$. 13-19

Figures S19-S21. HRESIMS of **1**-**3**. 20-22

Figure S1. ^{1}H NMR (500 MHz) Spectrum of Compound **1** in $CDCl_3$

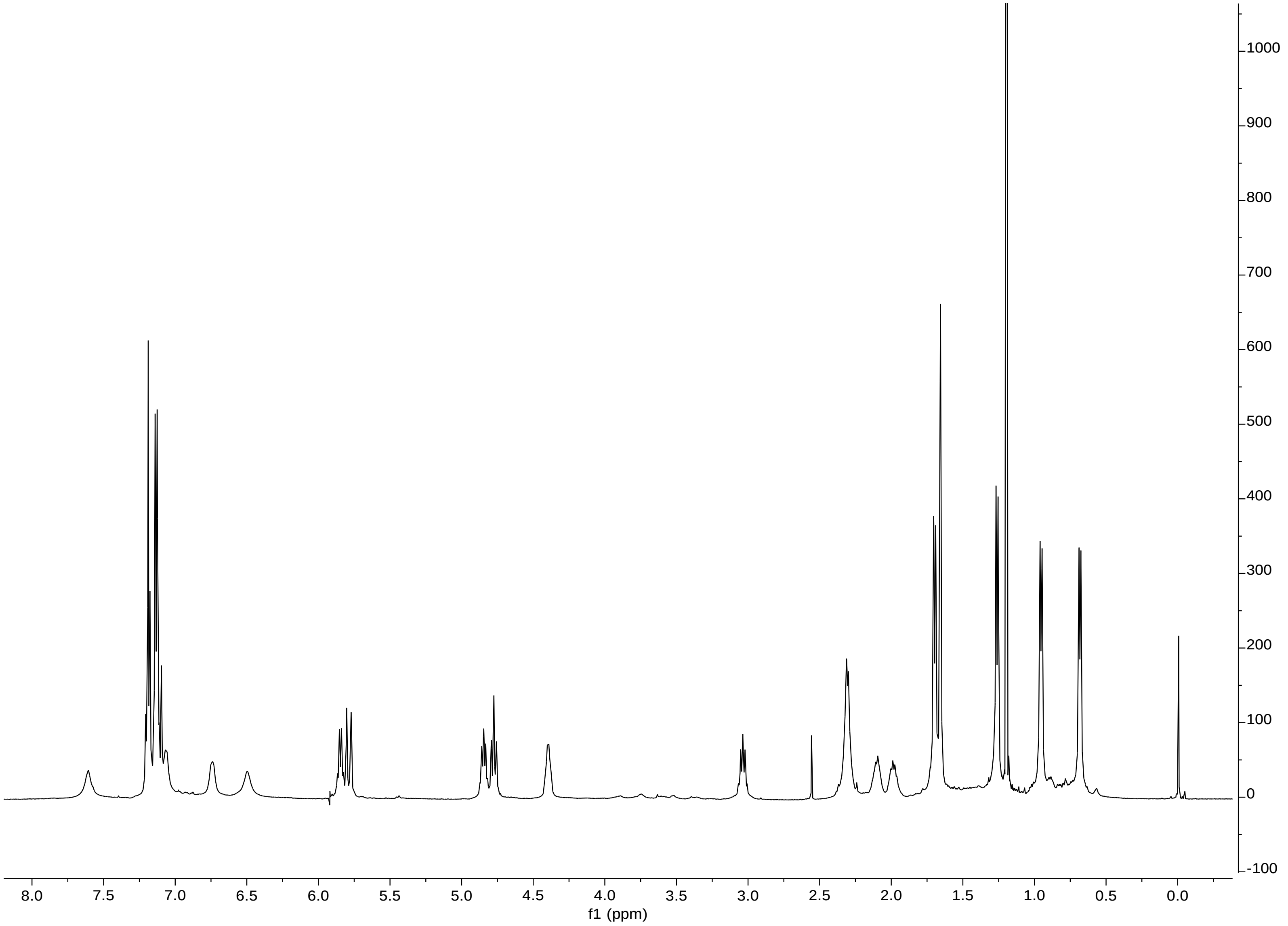

Figure S2. gCOSY (500 MHz) Spectrum of Compound **1** in $CDCl_3$

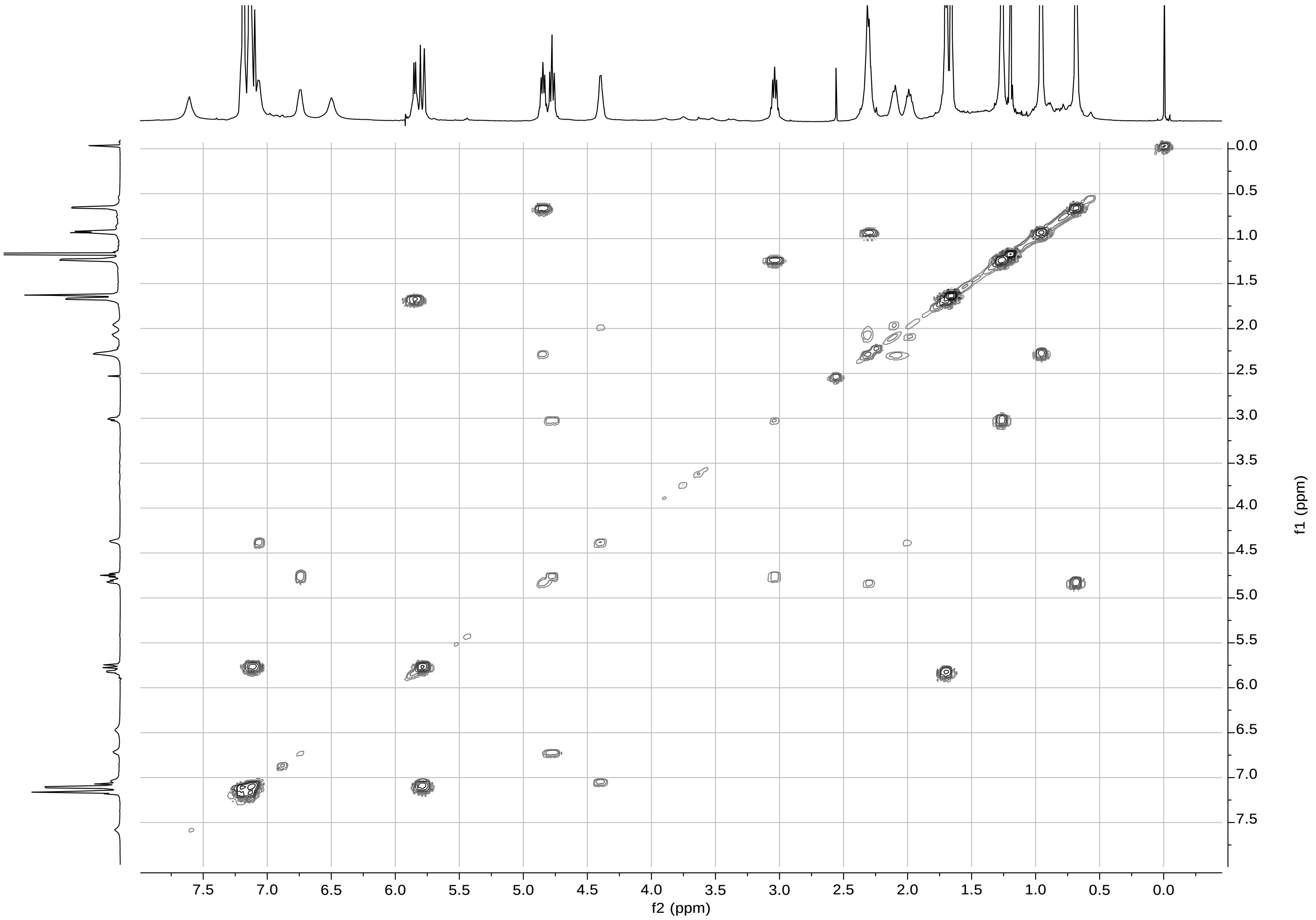

Figure S3. gHSQC (500 MHz) Spectrum of Compound **1** in $CDCl_3$

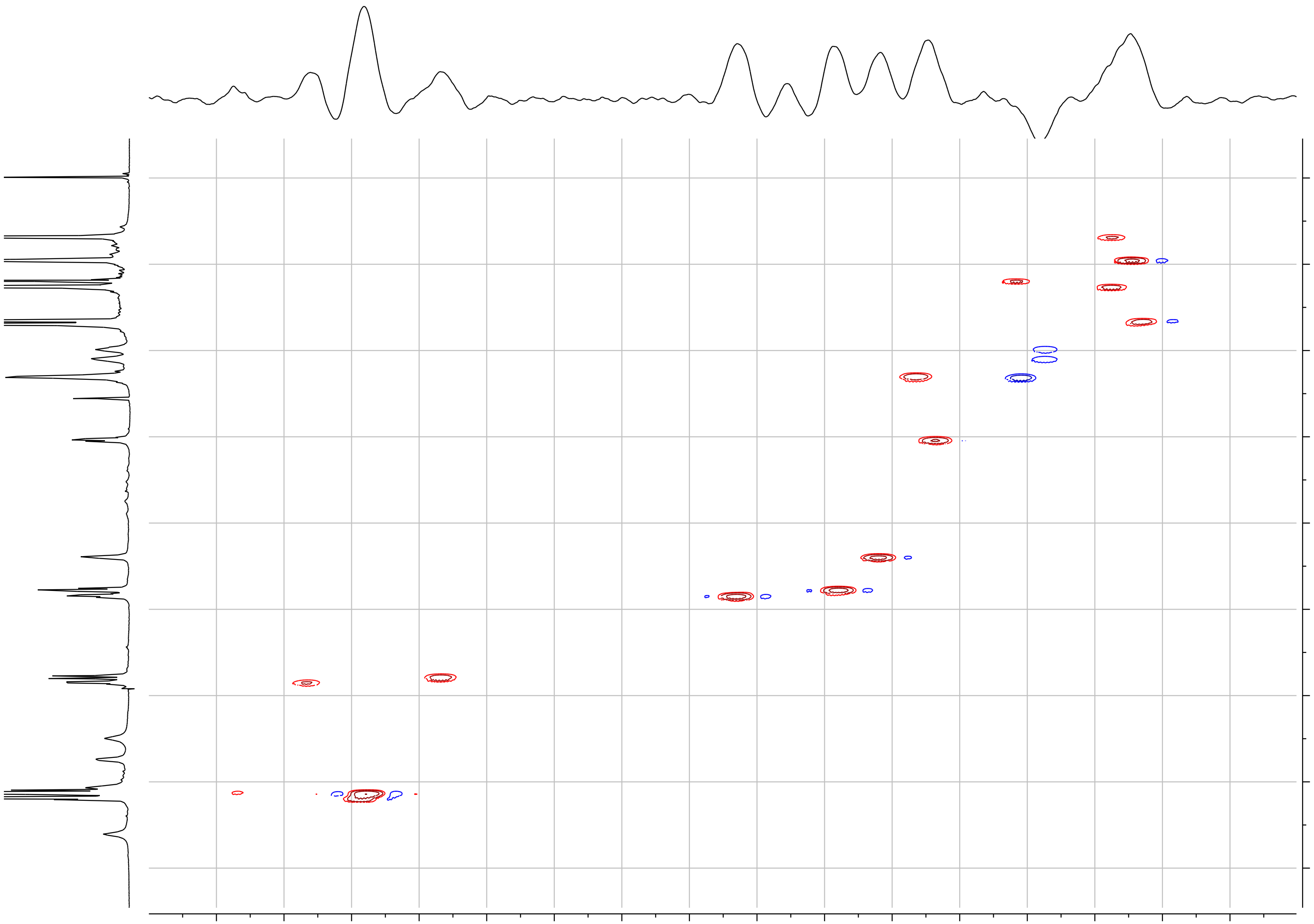

Figure S4. gHMBC (500 MHz) Spectrum of Compound **1** in $CDCl_3$

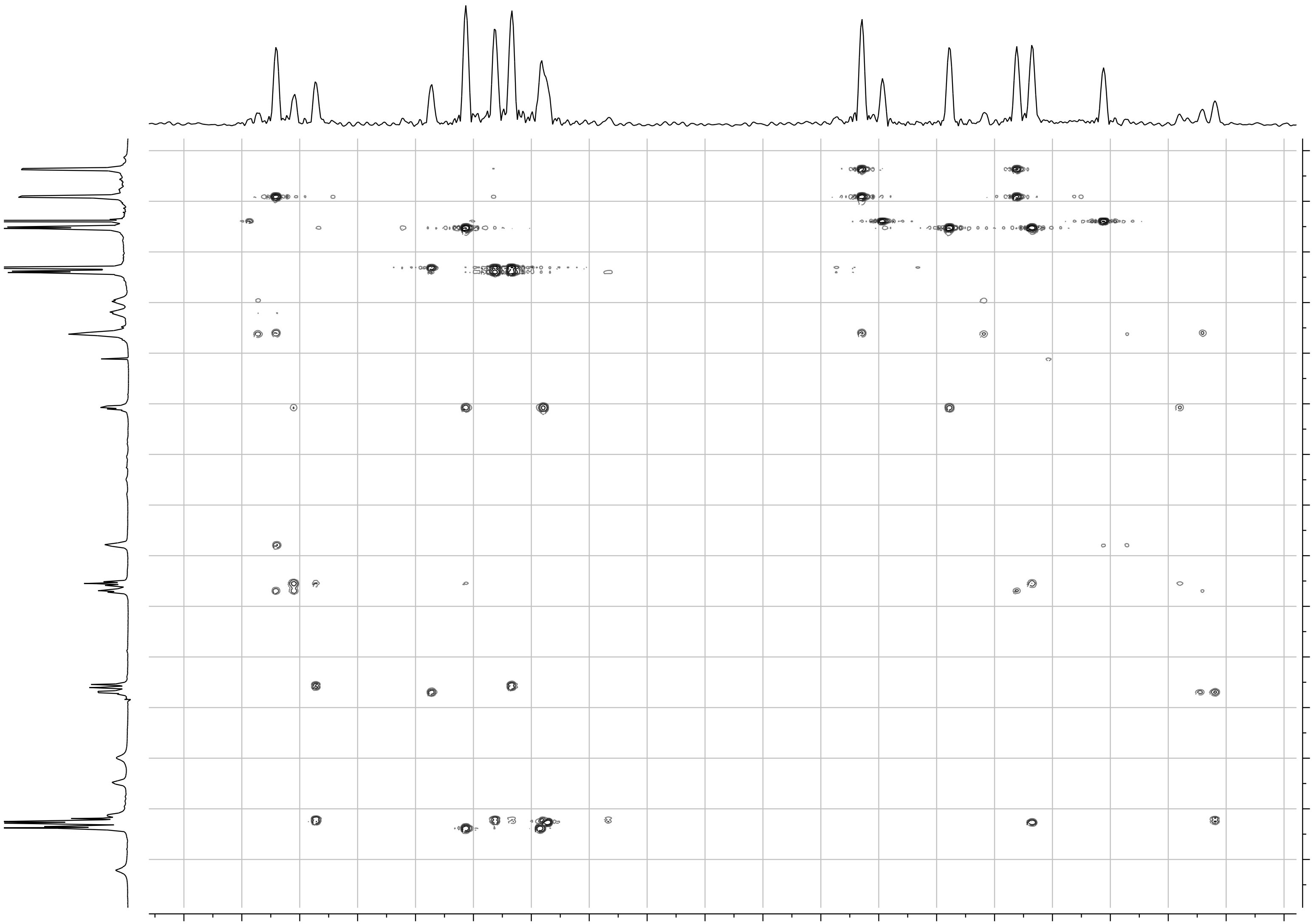

Figure S5. ROESY (500 MHz) Spectrum of Compound **1** in $CDCl_3$

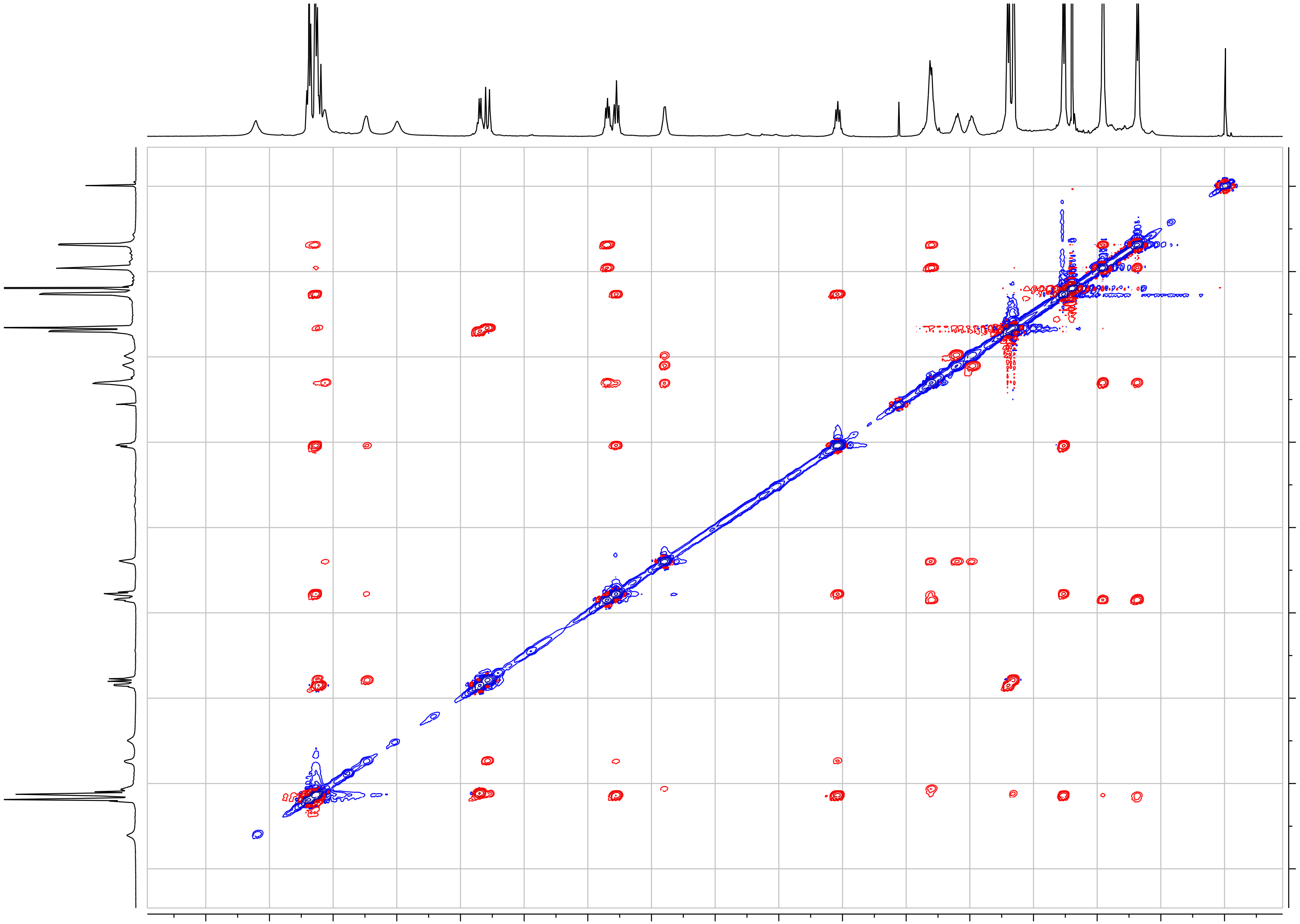

Figure S6. ^{1}H NMR (400 MHz) Spectrum of Compound **2** in $CDCl_3$

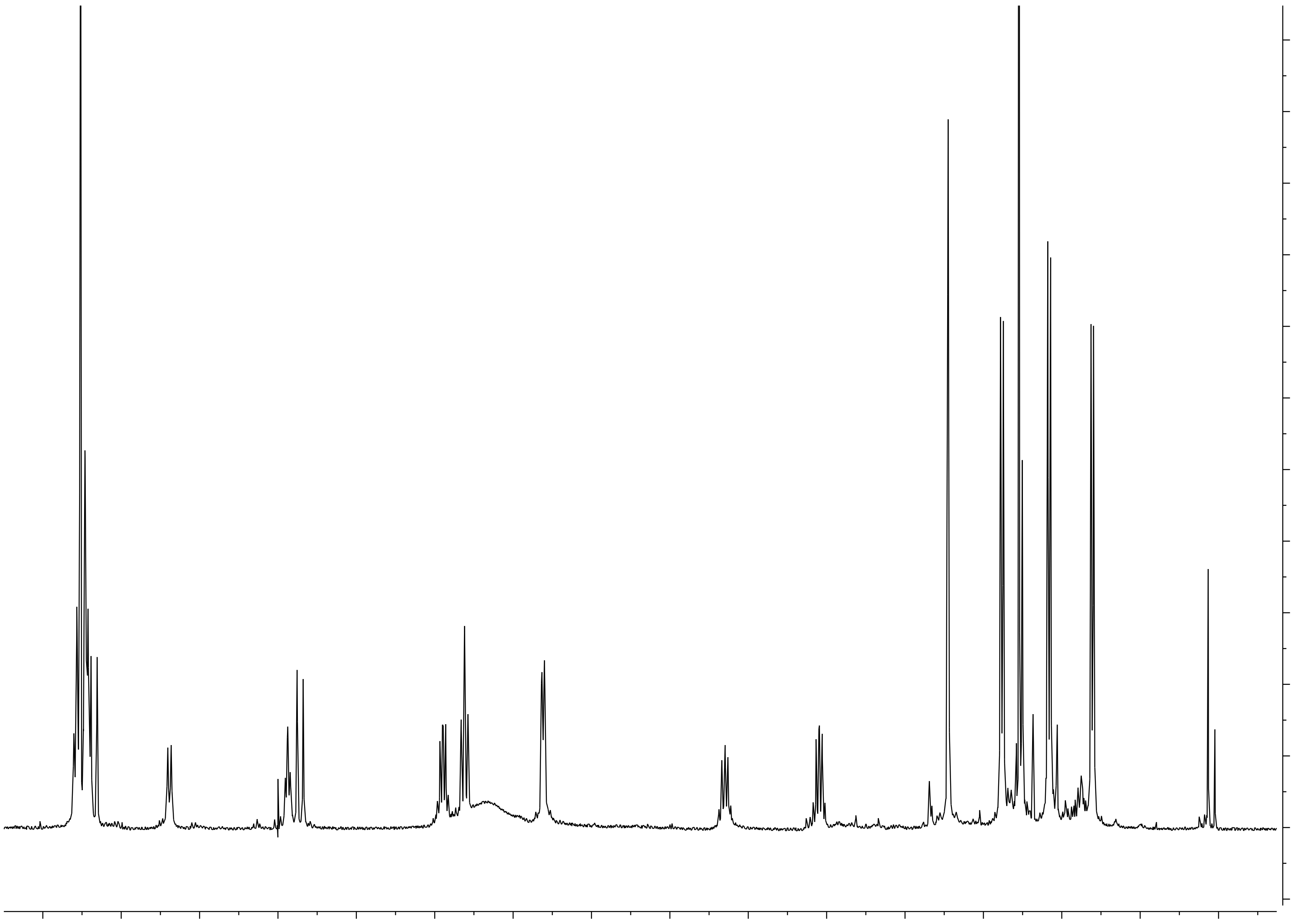

Figure S7. ^{13}C NMR (100 MHz) Spectrum of Compound **2** in $CDCl_3$

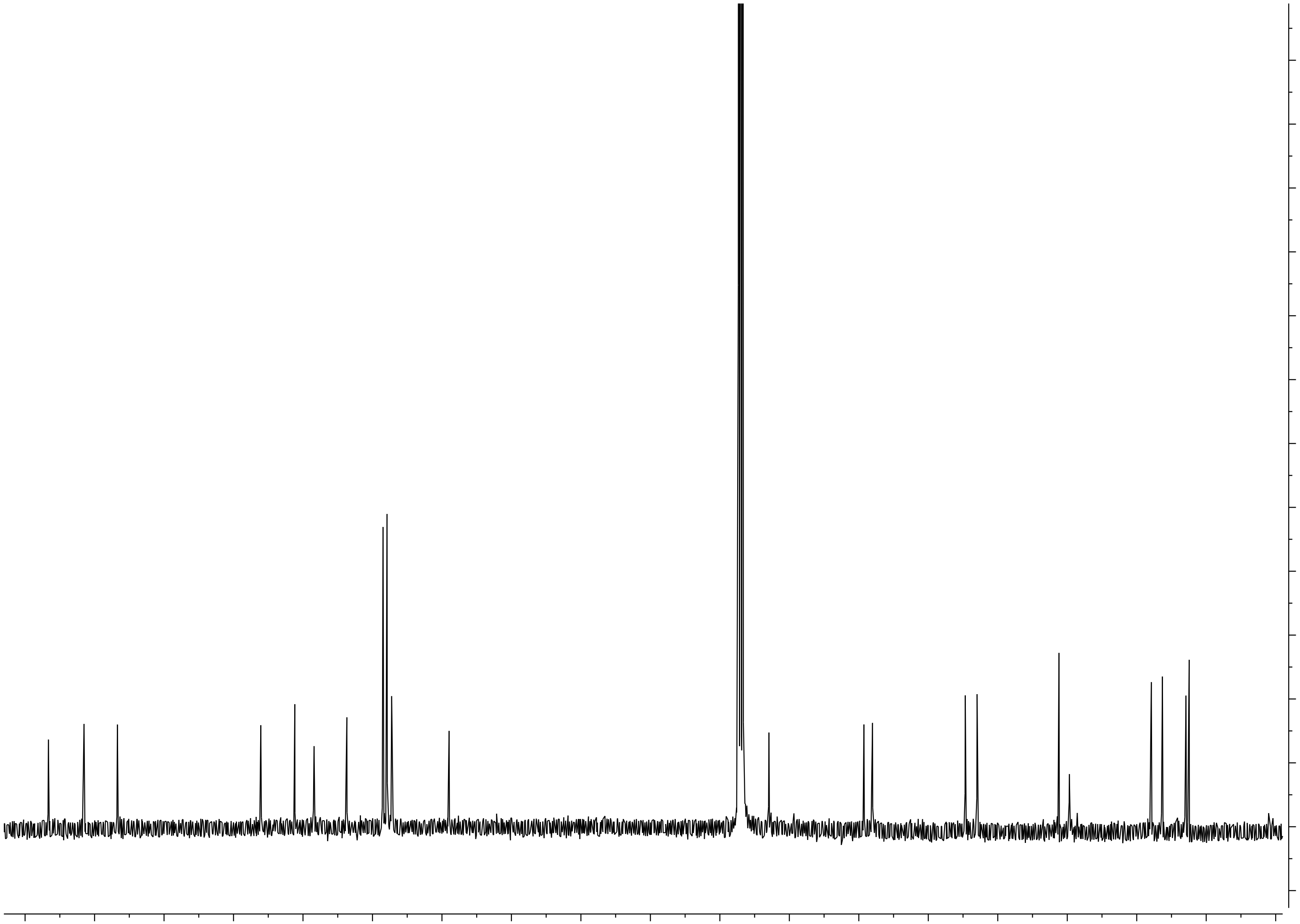

Figure S8. gCOSY (400 MHz) Spectrum of Compound **2** in $CDCl_3$

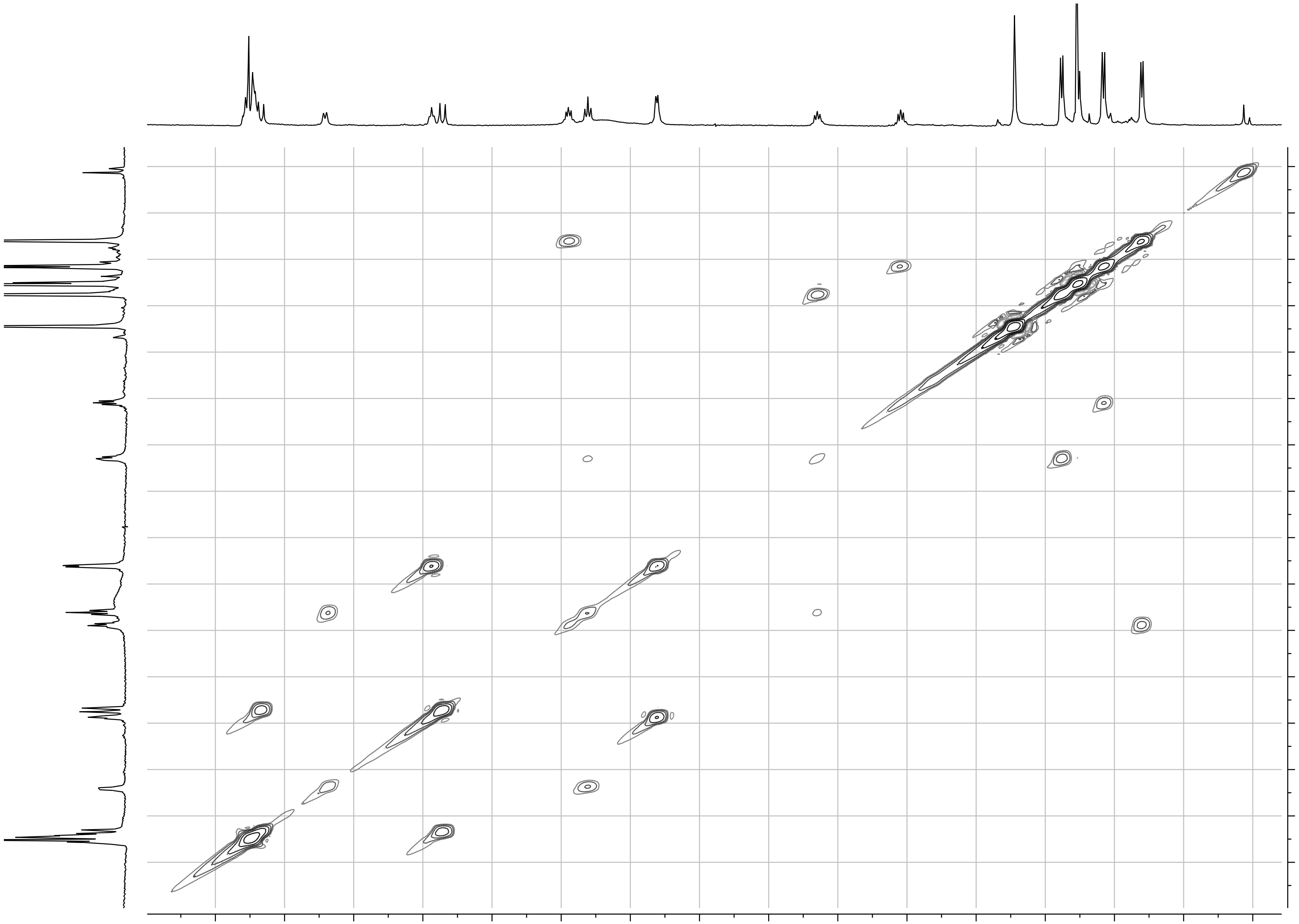

Figure S9. TOCSY (400 MHz) Spectrum of Compound **2** in $CDCl_3$

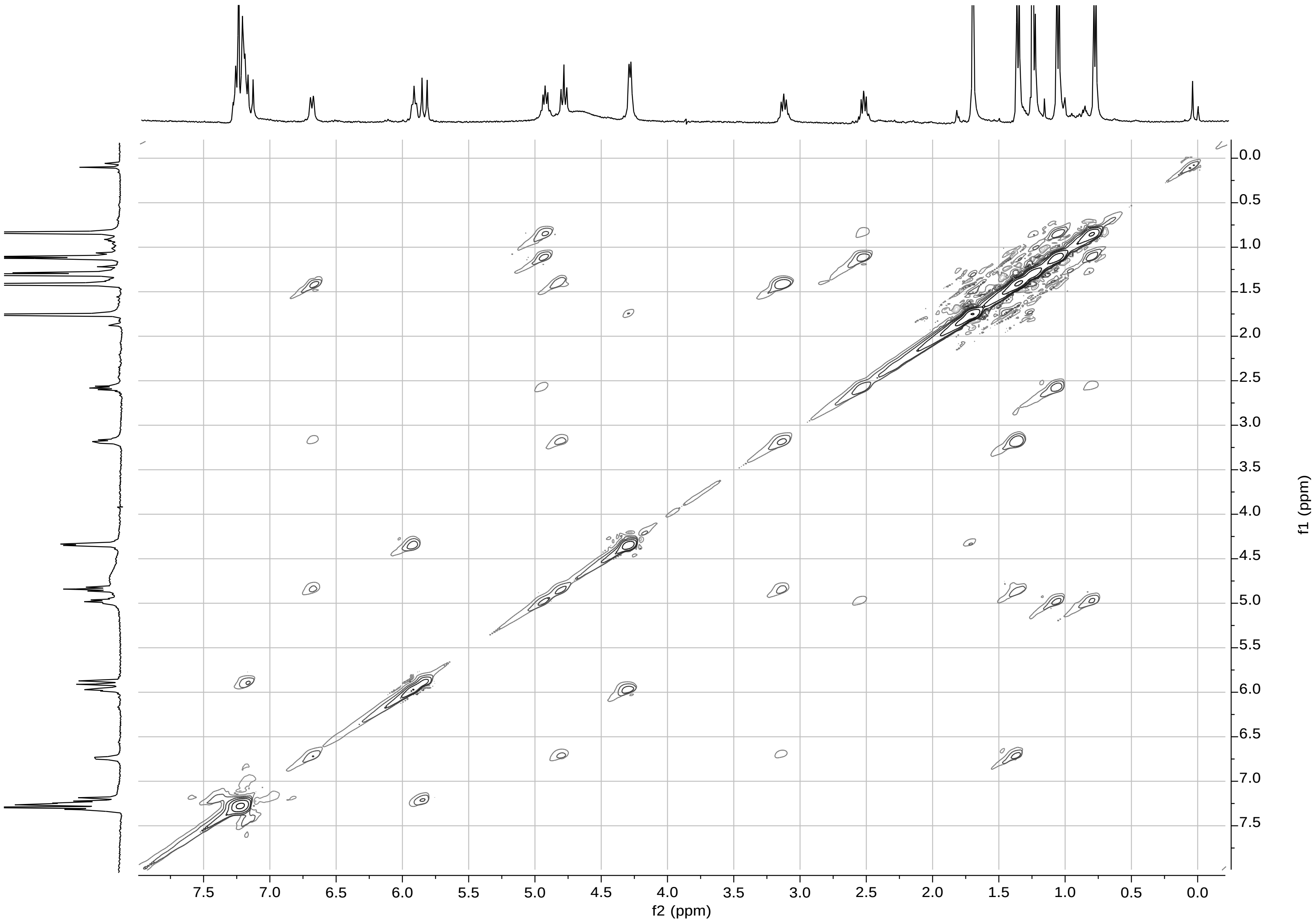

Figure S10. gHSQC (400 MHz) Spectrum of Compound **2** in $CDCl_3$

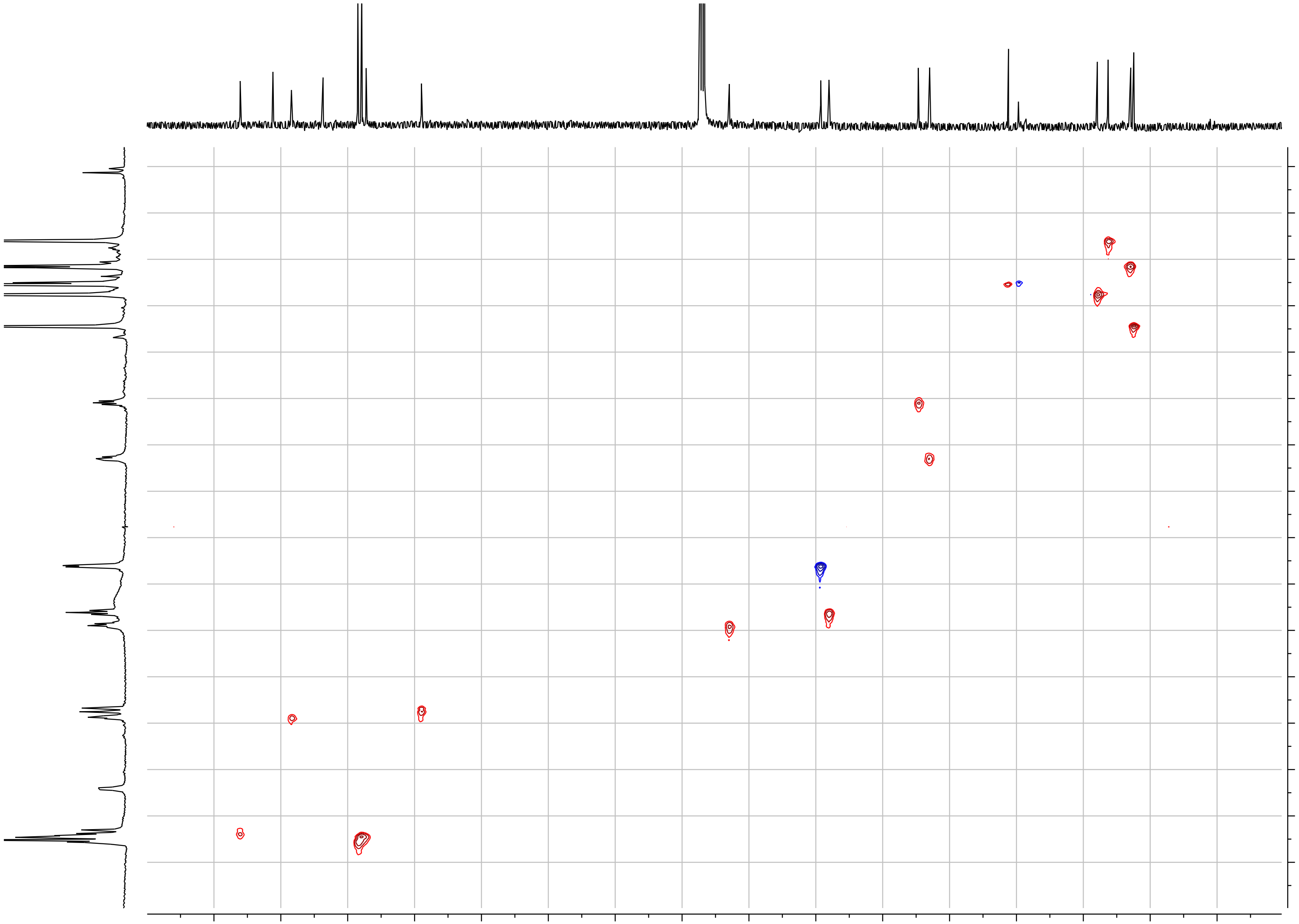

Figure S11. gHMBC (400 MHz) Spectrum of Compound **2** in $CDCl_3$

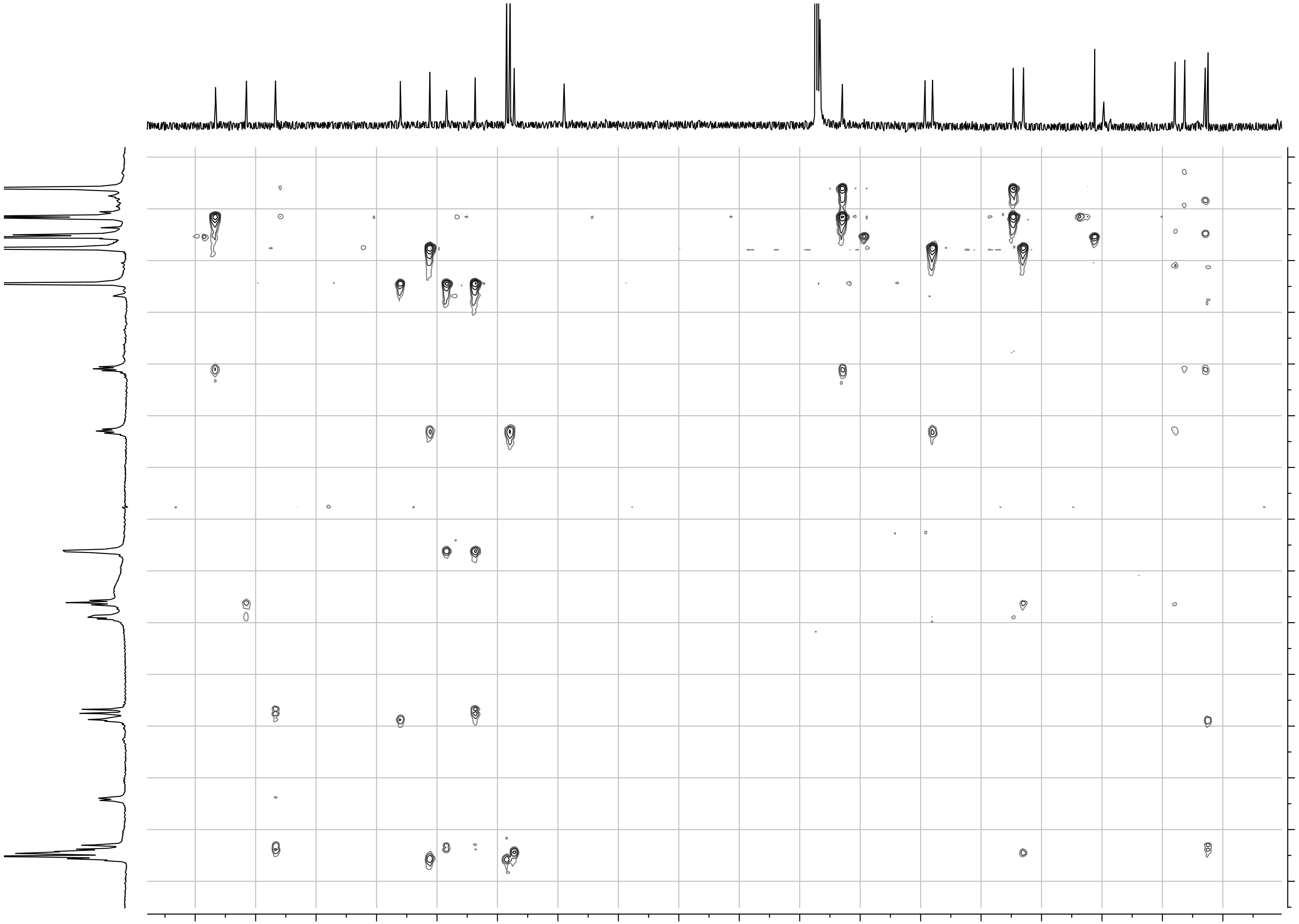

Figure S12.^{1}H NMR (500 MHz) Spectrum of Compound **3** in $CDCl_3$

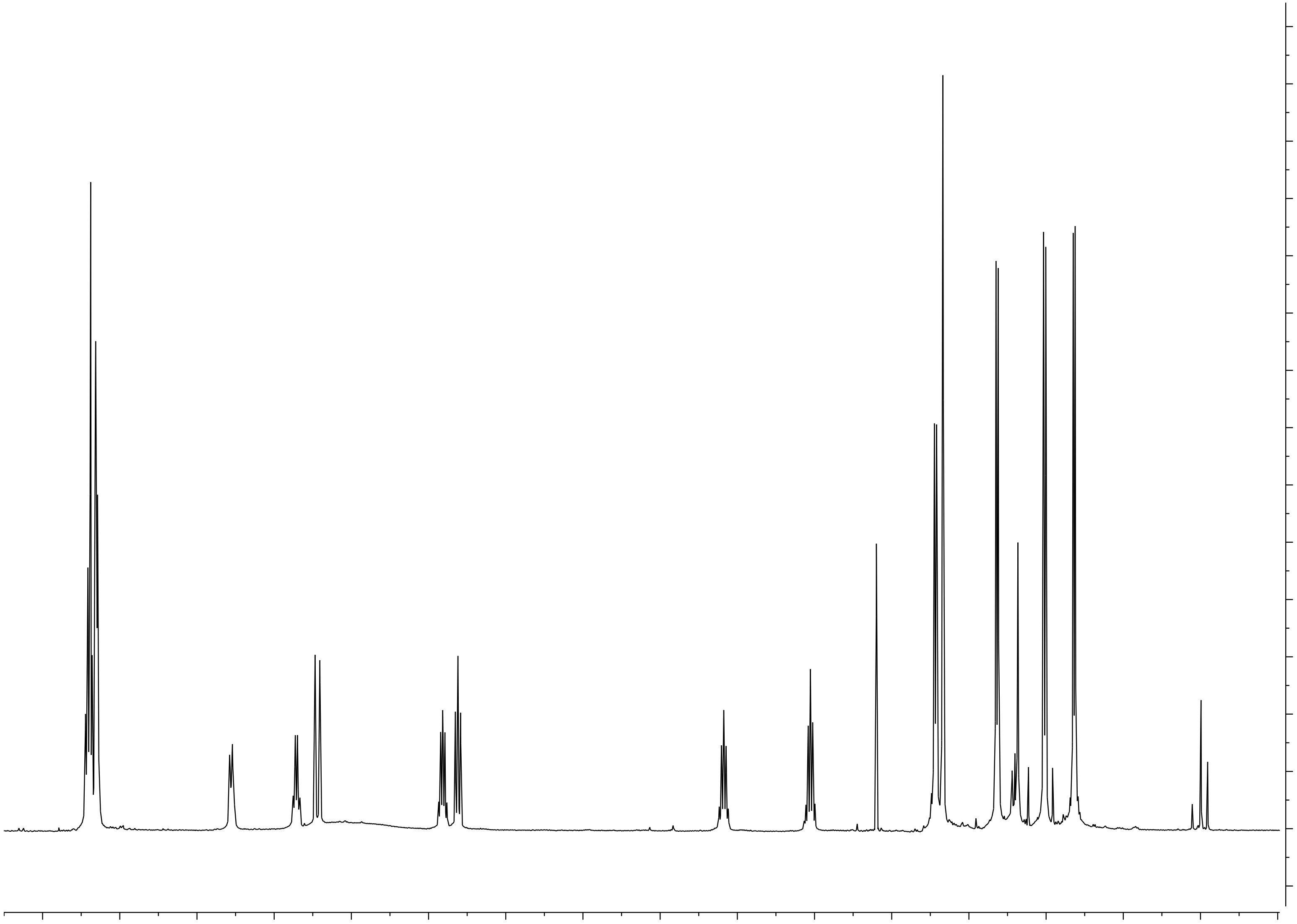

Figure S13. ^{13}C NMR (100 MHz) Spectrum of Compound **3** in $CDCl_3$

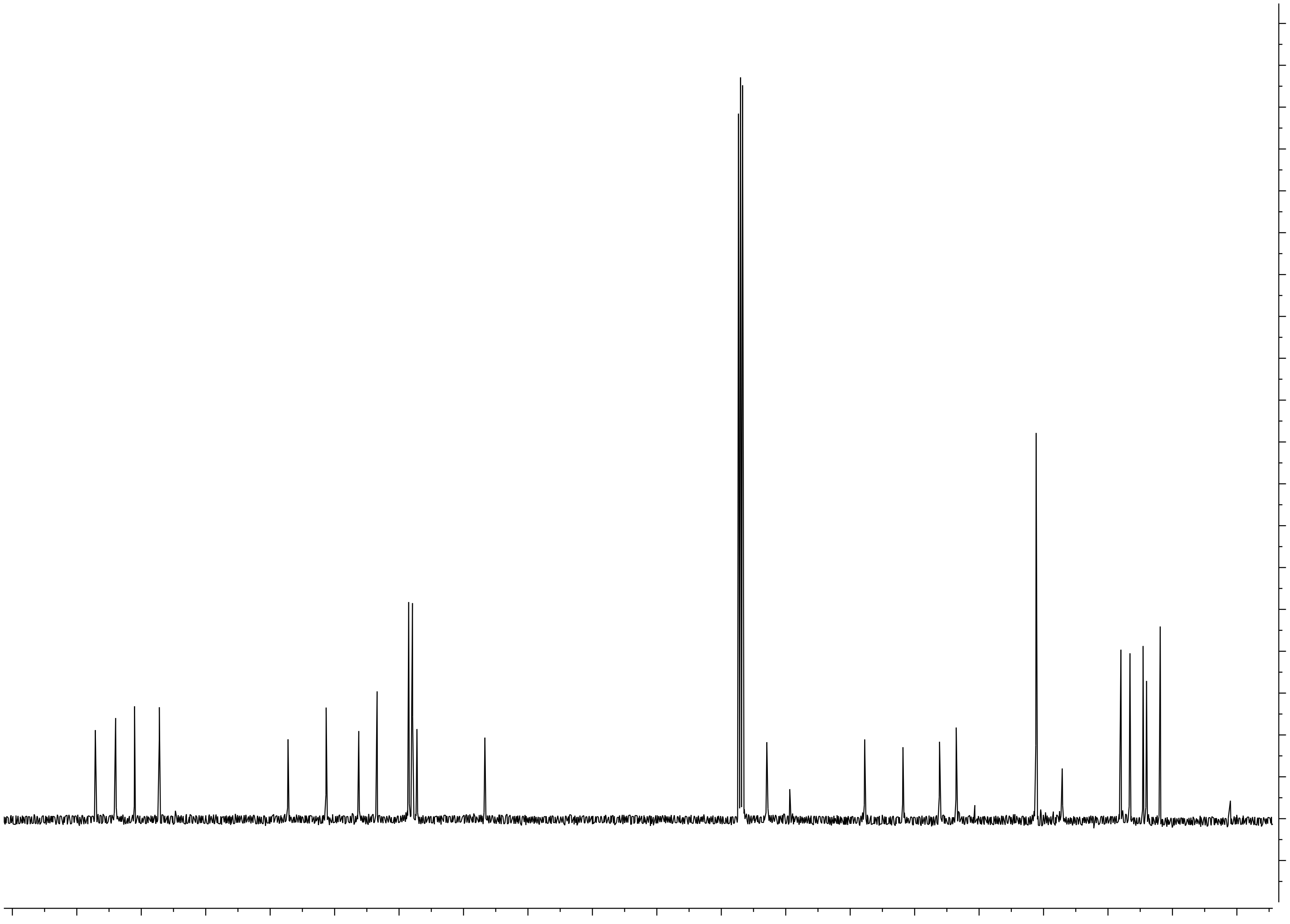

Figure S14.gCOSY (500 MHz) Spectrum of Compound **3** in $CDCl_3$

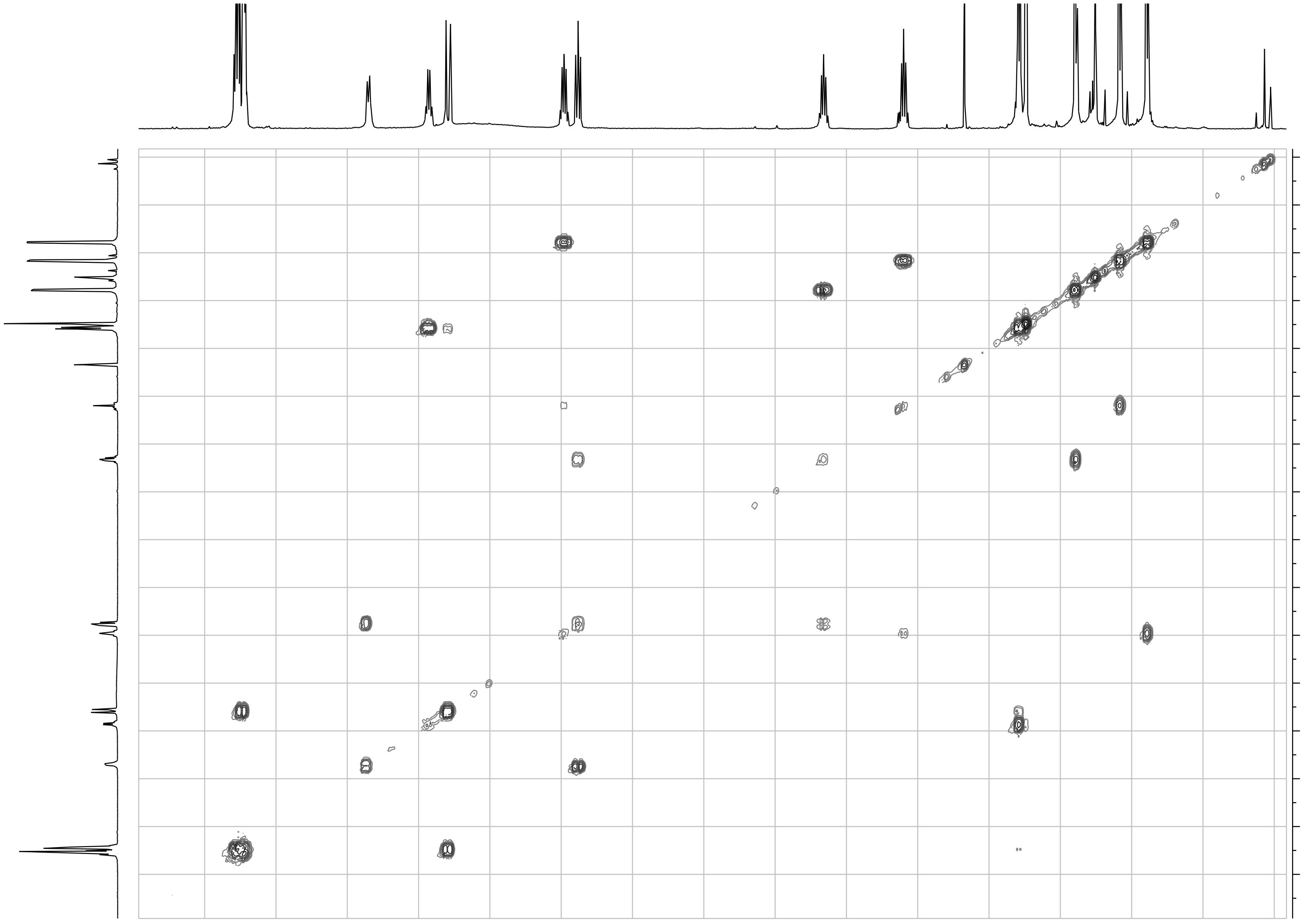

Figure S15. TOCSY (500 MHz) Spectrum of Compound 3 in $CDCl_3$

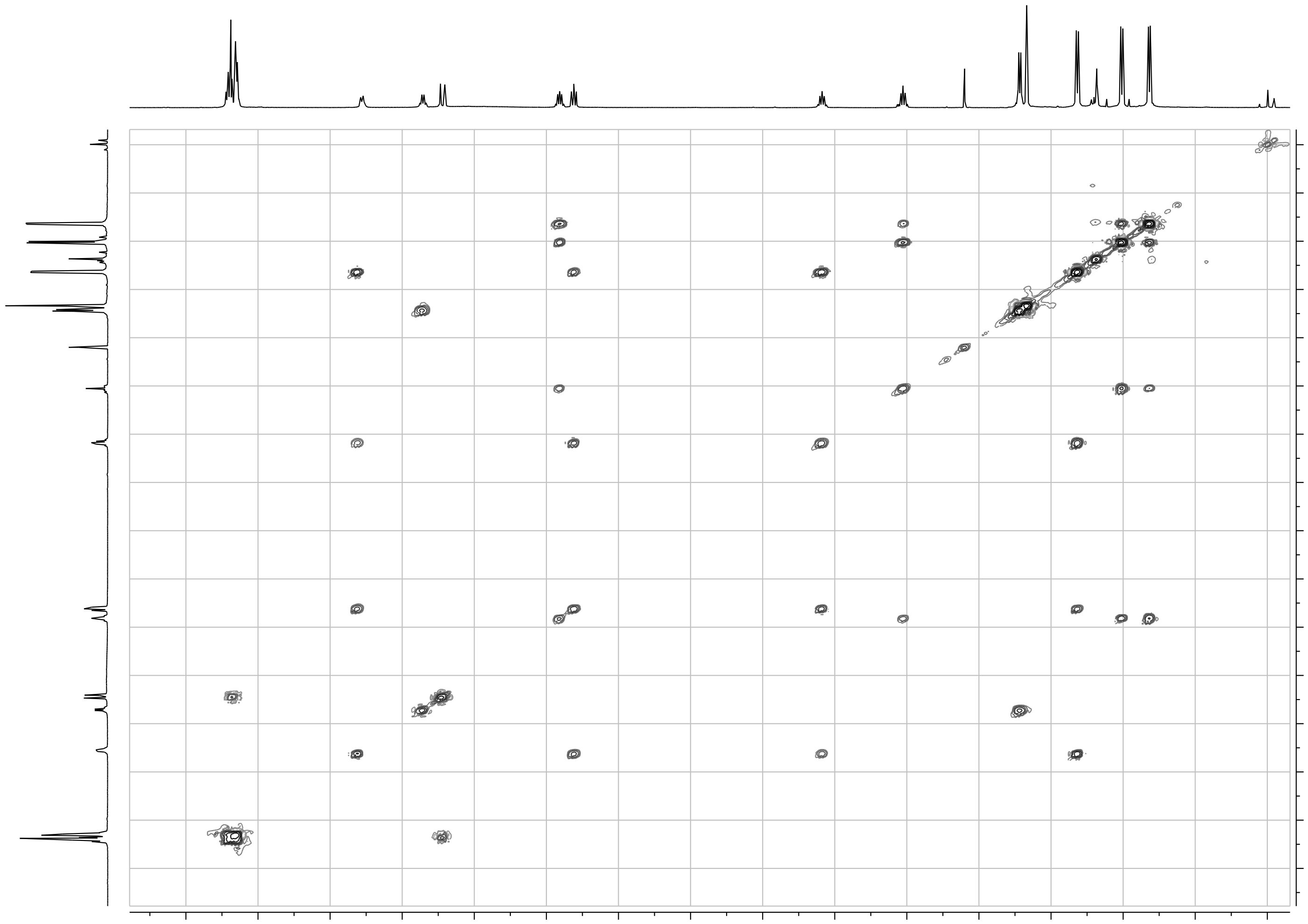

Figure S16. gHSQC (500 MHz) Spectrum of Compound **3** in $CDCl_3$

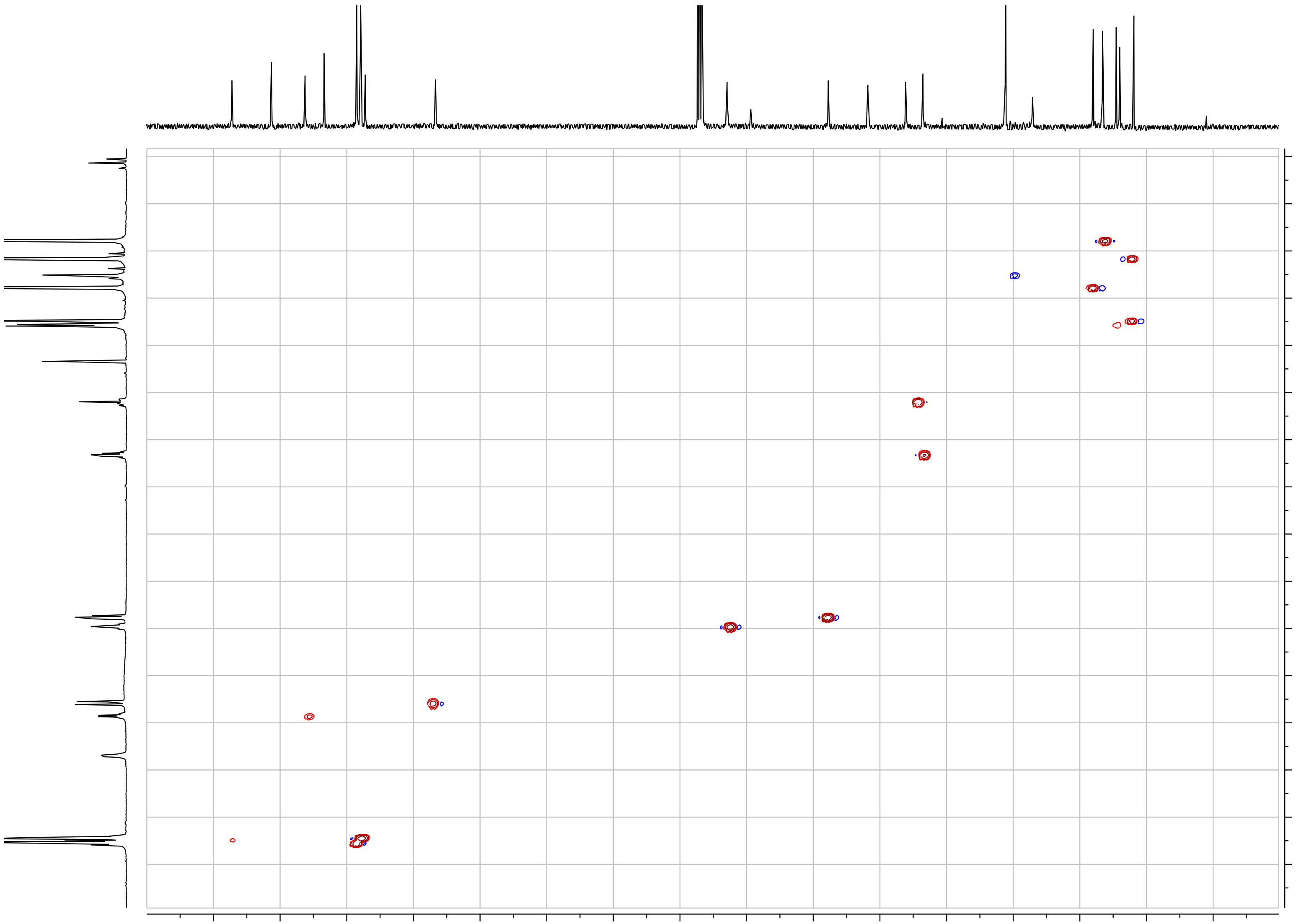

Figure S17.gHMBC (500 MHz) Spectrum of Compound 3 in $CDCl_3$

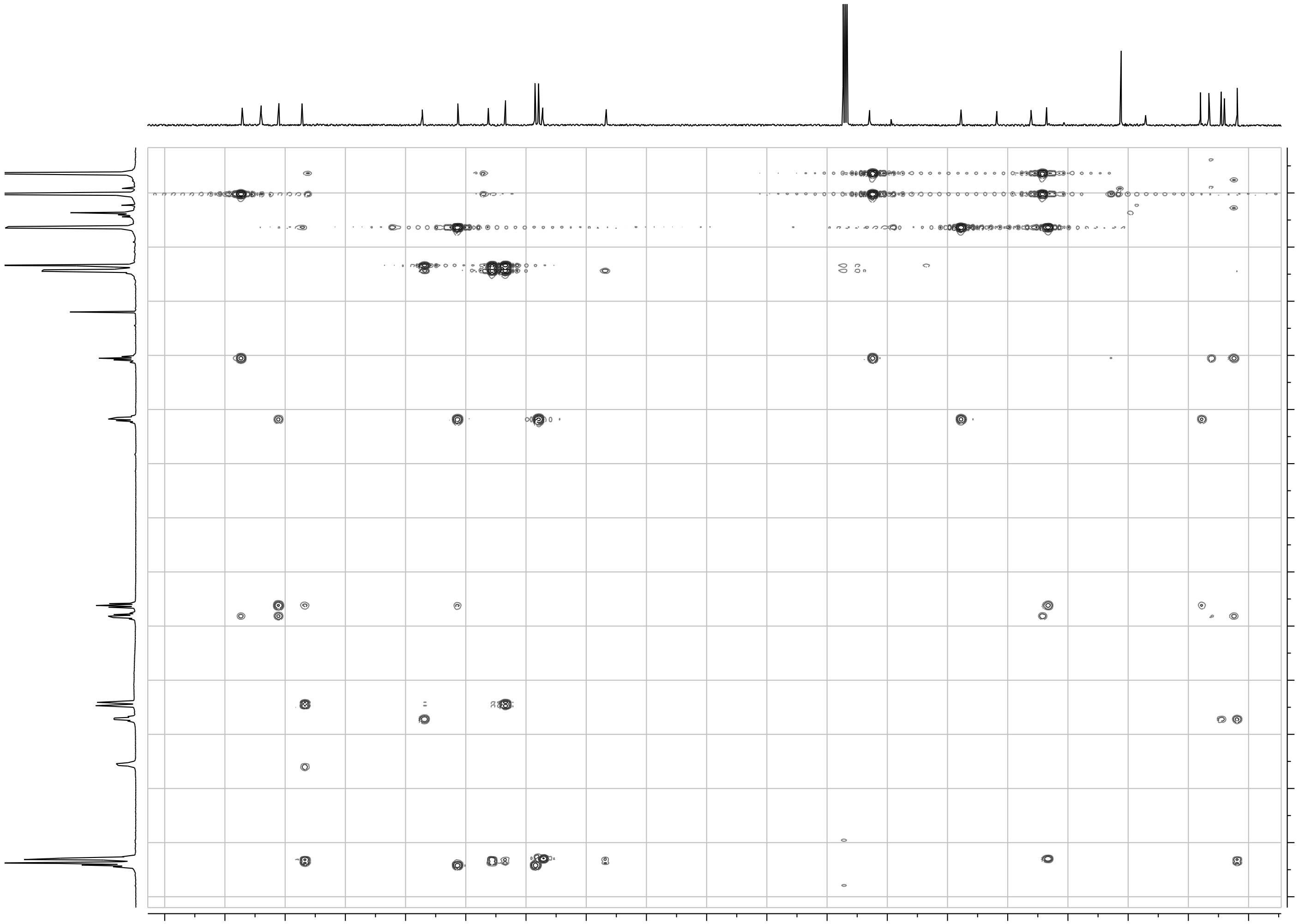

Figure S18.ROESY (500 MHz) Spectrum of Compound 3 in $CDCl_3$

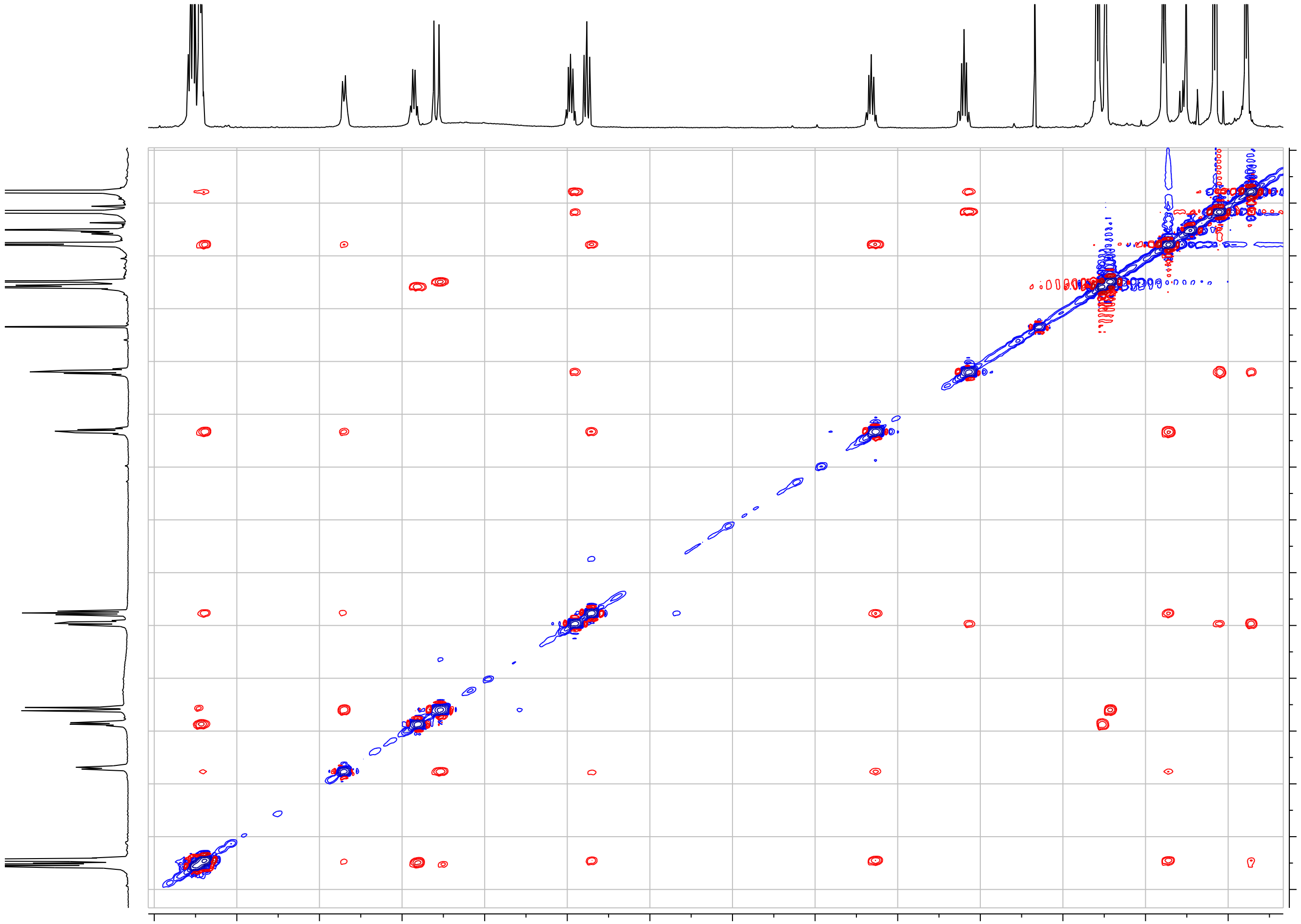

Figure S19. HRESIMS *m/z*516.2709 (calcd for $C_{27}H_{38}N_3O_7$516.2704) of 1.

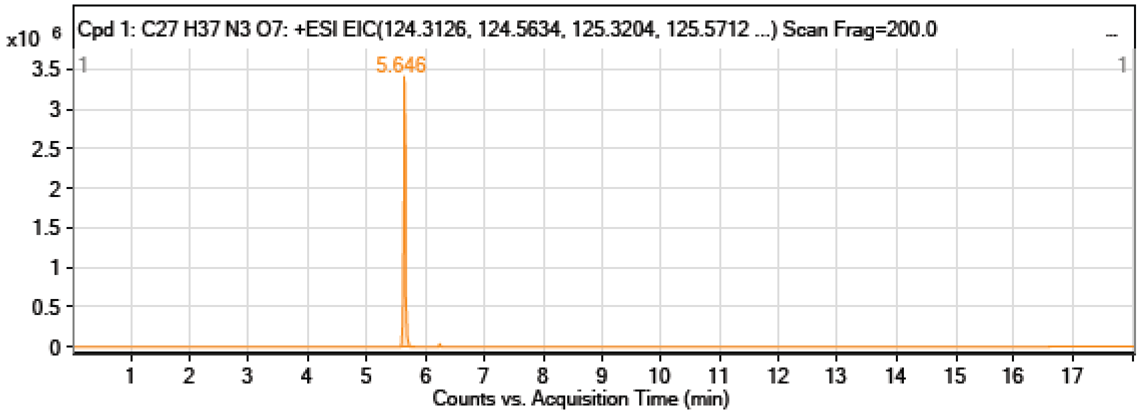

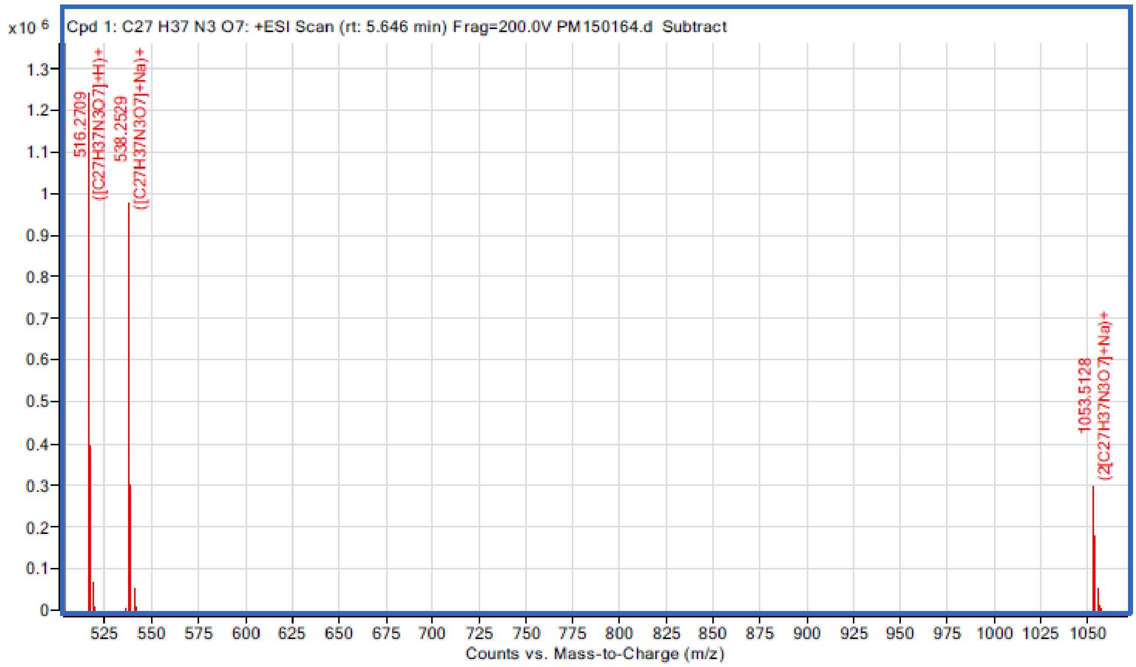

Figure S20. HRESIMS *m/z*404.2099 (calcd for $C_{22}H_{30}NO_6$404. 2079) of 2.

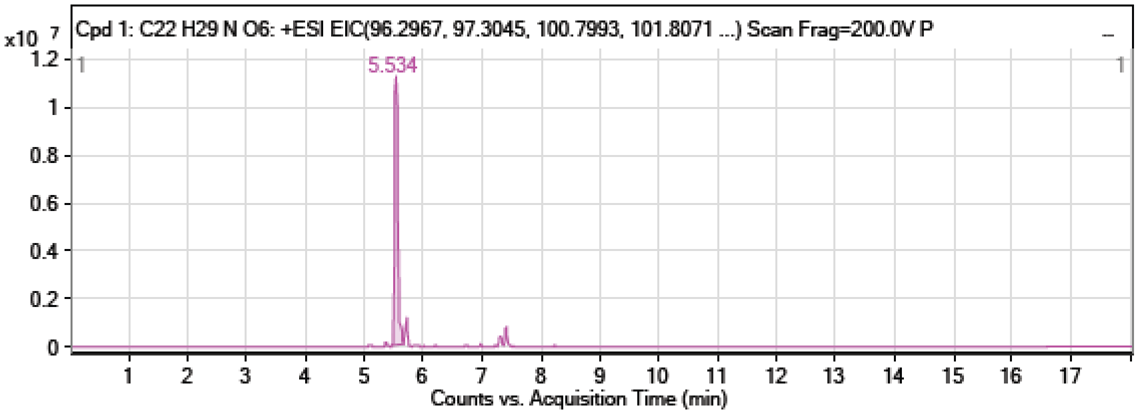

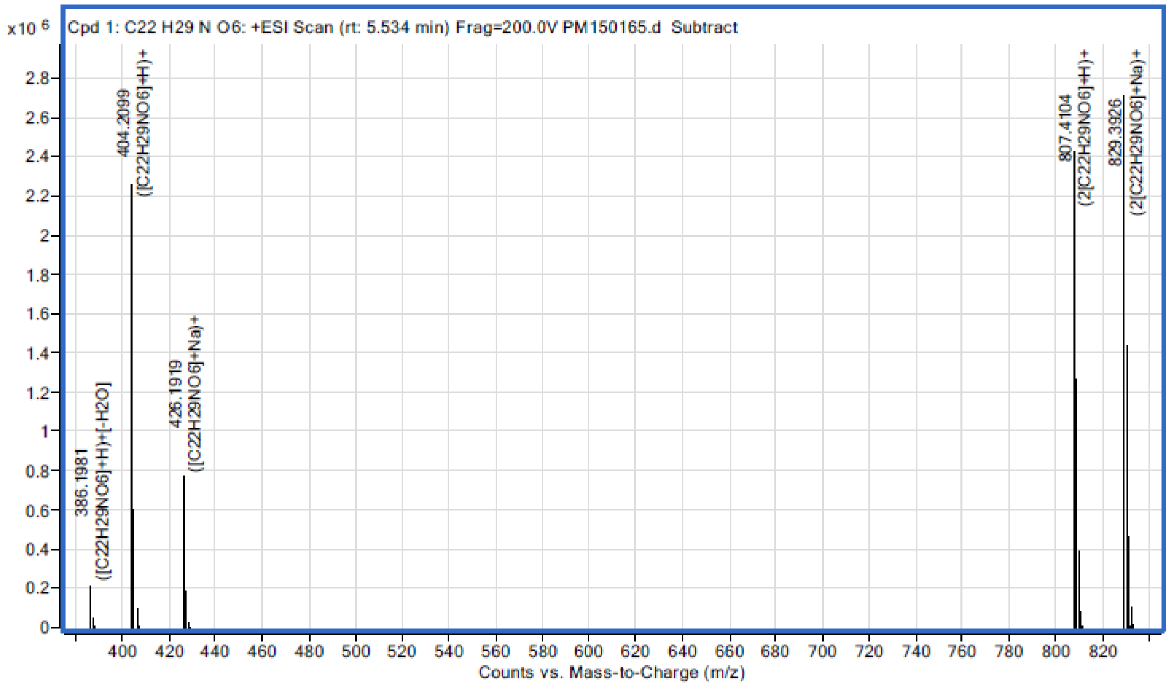

Figure S21. ESIMS *m/z*388.2141 (calcd for$C_{22}H_{30}NO_5$ 388.2118) of 3.

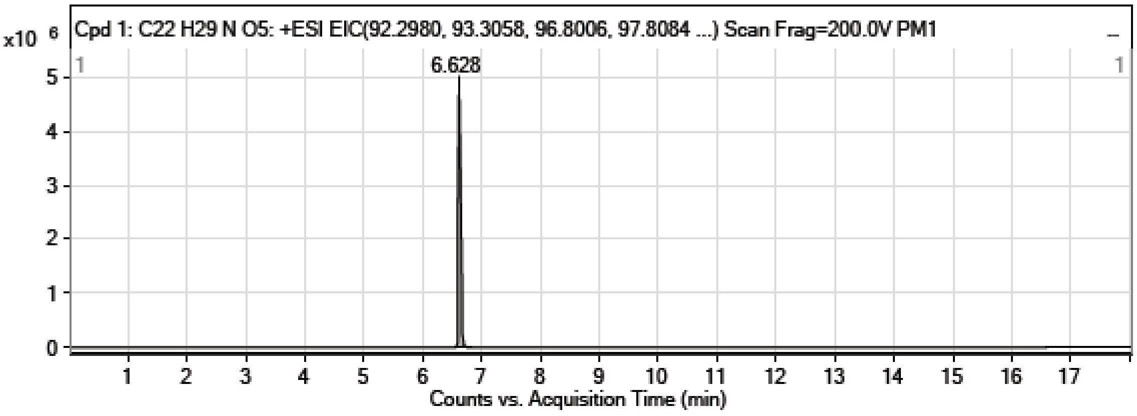

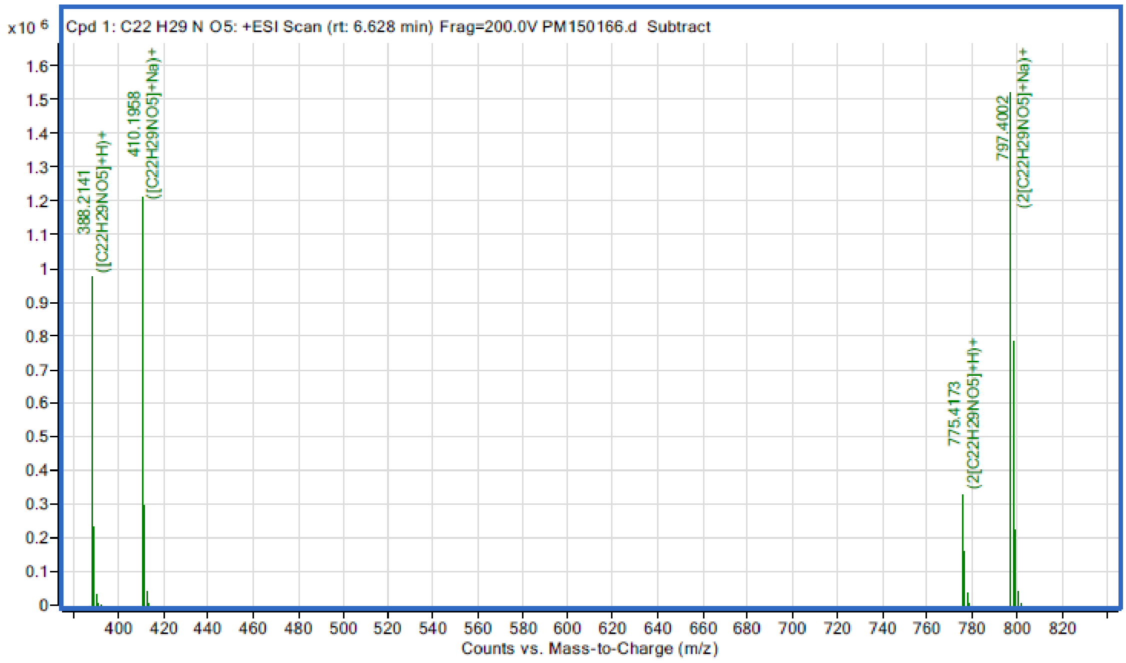

Supplement: Supplementary file 1 [file marinedrugs-16-00259-s001.zip › Supplementary file 1.pdf]
