# Supplementary material for: Characterization of the Jomthonic Acids Biosynthesis Pathway and Isolation of Novel Analogues in Streptomyces caniferus GUA-06-05-006A
Source: Mar Drugs. 2018 Jul 31;16(8):259. doi: 10.3390/md16080259 (PMC6117699; doi:10.3390/md16080259)
Supplement: Supplementary file 1 [file marinedrugs-16-00259-s001.zip › Supplementary file 2.pdf]

Figure S22. Alignment of JomP1 AT_2 conserved sequences with other acyltransferase domains. StiA_AT1, StiA_AT2 and StiC_AT: stimatellin biosynthesis [1] and MxaF_AT1 and MxaF_AT2: myxalamid biosynthesis in *S. aurantica* Sga15 [2].

```
JomP2_AT    GQASQY...GHSVGE...HAFH
StiC_AT     GQGSQY...GHSVGE...HAFH
JomP1_AT2   GQGGHT...GHSAGE...YPSH
MxaF_AT1    GHGSQW...GHSIGE...VAVH
StiA_AT1    GTGSQW...GHSIGE...TPSH
JomP1_AT1   GQGSQW...GHSLGE...VASH
StiA_AT2    GQGSQW...GHSMGE...VASH
MxaF_AT2    GQGSQW...GHSMGE...VASH
Consensus   GqgSQw...GHSxGE
```

Figure S23. Genetic complementation of mutant *ΔjomM*. UPLC analysis of jomthonic acids production in ΔjomM and complemented strain ΔM-pSEJomM.

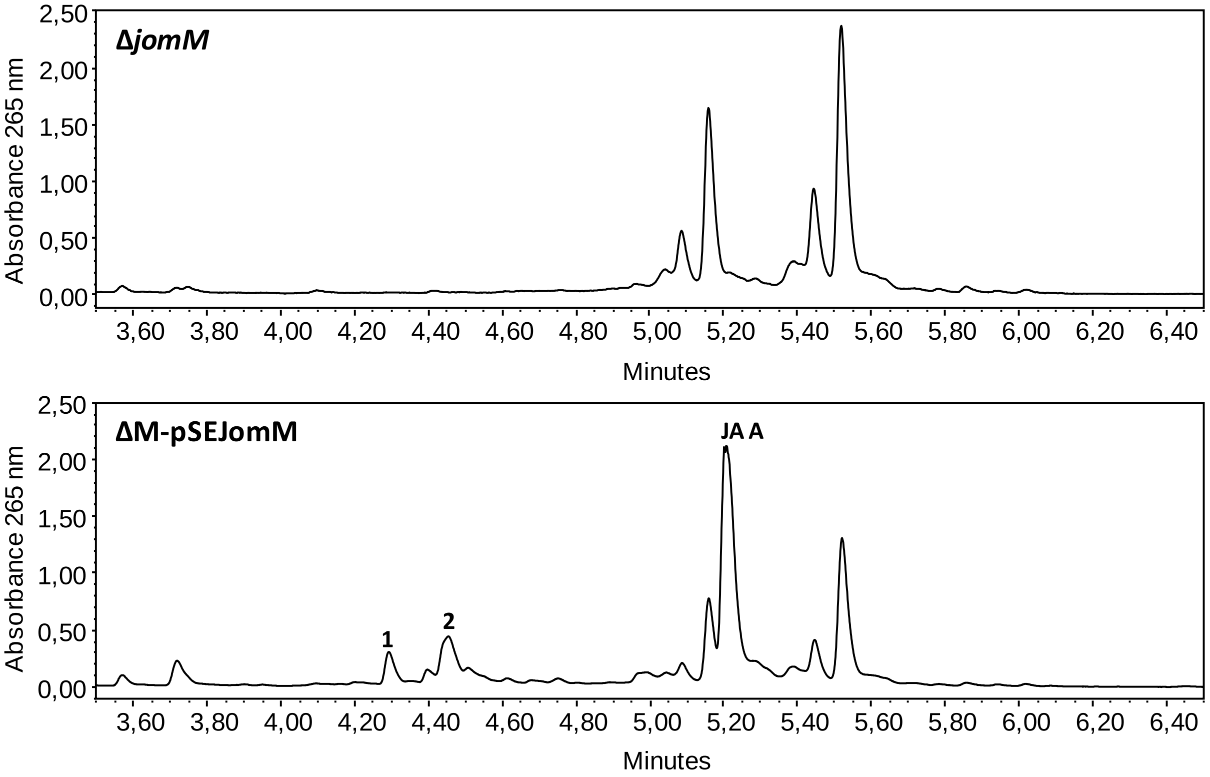

Figure S24. Mass spectra of compounds 1^a-2^a.

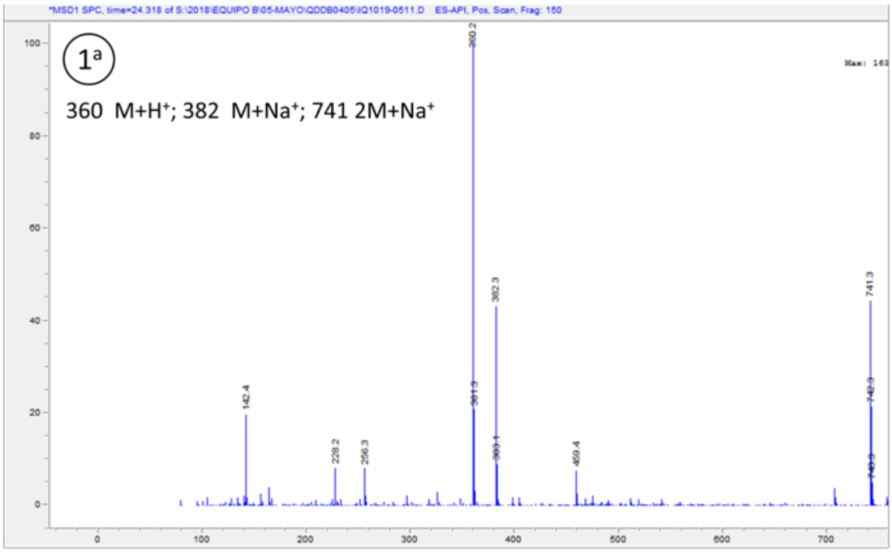

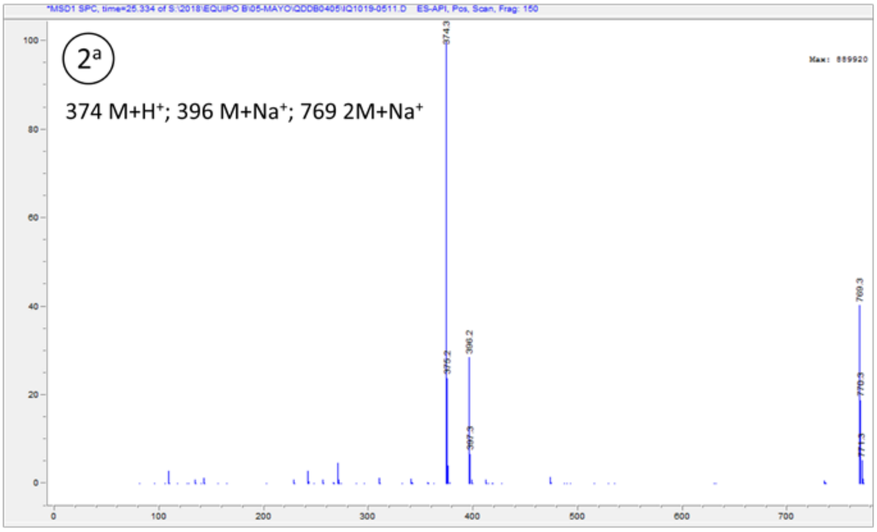

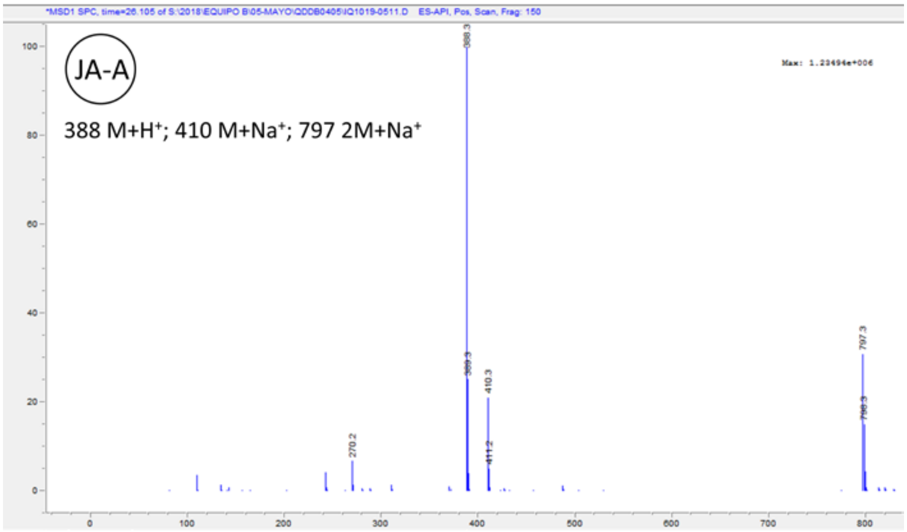

Figure S25. Identification of additional potential jomthonic acids. A) UPLC analysis of 5-fold concentrated extracts from G-p*ermE**. B) Mass spectra of four additional compounds with jomthonic acid absorption spectra. Based on mass spectra, a tentative chemical structure is suggested for compound **b** and **d**.

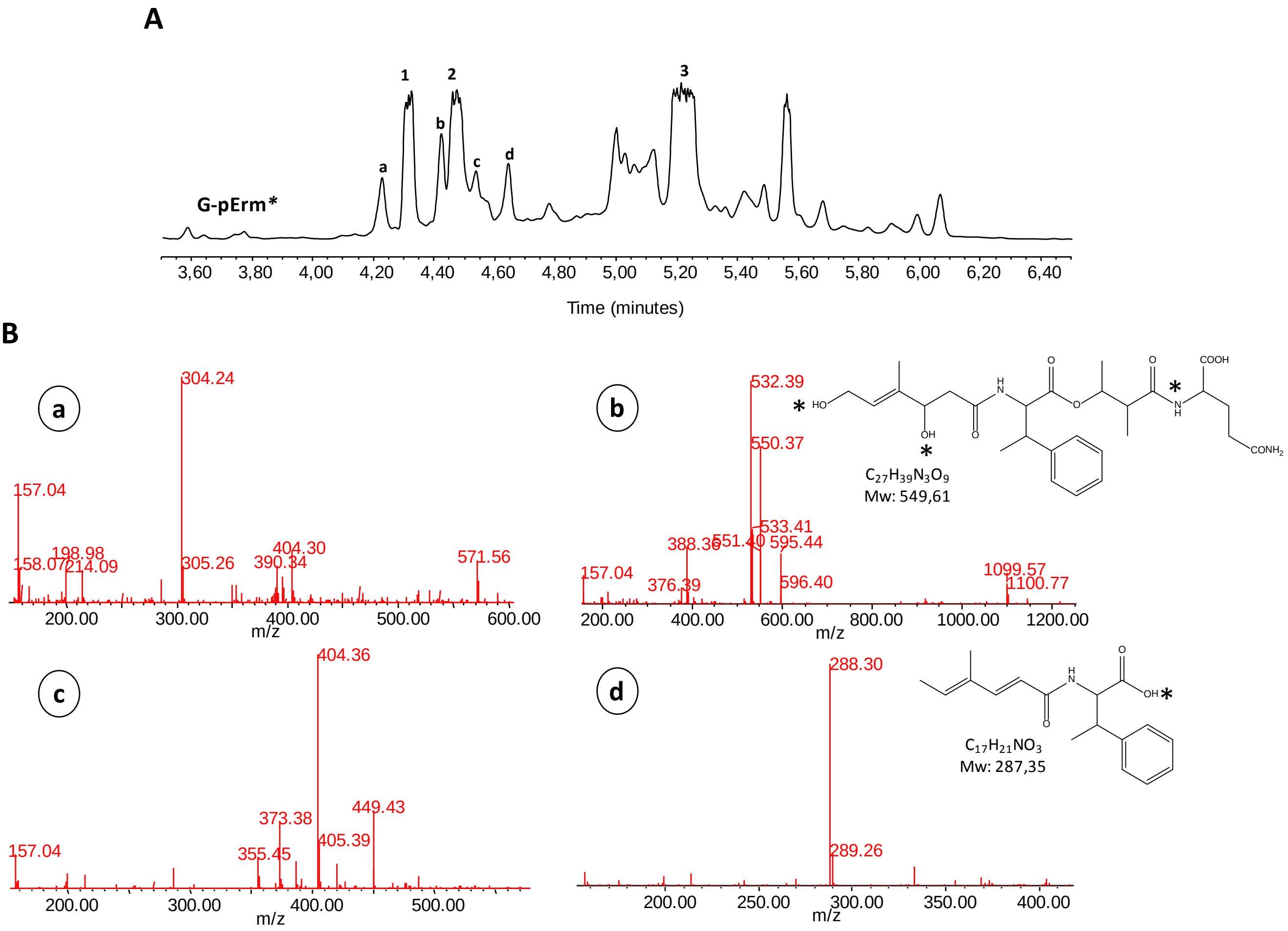

Figure S26. Alternative biosynthesis model of the JAs polyketide chain. JomP1AT$_1$ transfer the starting unit malonil-CoA and AT$_2$ catalizes an extension with methylmalonil-CoA. A dehydration reaction (+DH) is required to achieve the final structure of the polyketide chain present in JA A.

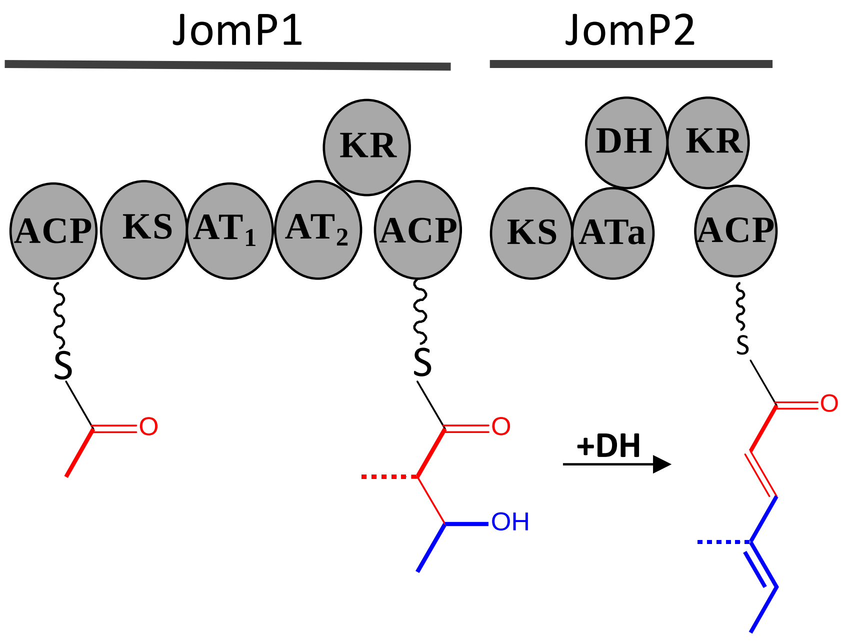

References

1. Gaitatzis, N.; Silakowski, B.; Kunze, B.; Nordsiek, G.; Blöcker, H.; Höfle, G.; Müller, R. The biosynthesis of the aromatic myxobacterial electron transport inhibitor stigmatellin is directed by a novel type of modular polyketide synthase. *J. Biol. Chem.* **2002**, *277*, 13082–90, doi:10.1074/jbc.M111738200.

2. Silakowski, B.; Nordsiek, G.; Kunze, B.; Blöcker, H.; Müller, R. Novel features in a combined polyketide synthase/non-ribosomal peptide synthetase: the myxalamid biosynthetic gene cluster of the myxobacterium Stigmatella aurantiaca Sga15. *Chem. Biol.* **2001**, *8*, 59–69.
