# Supplementary material for: Characterization of the Jomthonic Acids Biosynthesis Pathway and Isolation of Novel Analogues in Streptomyces caniferus GUA-06-05-006A
Source: Mar Drugs. 2018 Jul 31;16(8):259. doi: 10.3390/md16080259 (PMC6117699; doi:10.3390/md16080259)
Supplement: Supplementary file 1 [file marinedrugs-16-00259-s001.zip › Supplementary file 3.pdf]

Table S1. Primers and plasmids used in this work

Primer	Sequence (5'-3')	PCR product and purpose	Plasmid (related strain)
		***ermE*p* insertion**	
**EcoRI**-EP1	TATA**GAATTC**GCTGACTTCGAGGAAGTGGT	2769-bp fragment for *ermE*p* insertion upstream (-104 bp from start codon) of *jomP1*in chromosome	**pEjomP1** (G-pErm*)
**PstI**-EP1	TATA**CTGCAG**CCCGCAATTCAGGTTCTTTA		
		**Gene mutation**	
**EcoRI**-P1	TATA**GAATTC**GAGAGTTCTGCTTCGGGATG	2938-bp fragment for *jomP1* interruption	**pIjomP1** (*jomP1*-)
**HindIII**-P1	TATA**AAGCTT**GCGTCGCAGTACTTCGGTAT		
**EcoRI**-N	TATA**GAATTC**GCTGGTCGGTGTCCTCTC	2246-bp fragment for *jomN* interruption	**pIjomN** (*jomN*-)
**HindIII**-N	TATA**AAGCTT**GTCCTCCTACGACGCCTTG		
**NsiI**-orf8	TATA**ATGCAT**CTTCCGCATCAGAGGACAGT	2590-bp upstream flanking region for *orf8*deletion	**pDorf8** (Δorf8-araC)
**SpeI**-orf98	TATA**ACTAGT** GATGTTCCATTCGCCTGTCT		
**EcoRV**- orf8	TATA**GATATC**GGTCGGTACAACGTCGTCTT	2433-bp downstream flanking region for *orf8* deletion	
**BamHI**- orf8	TATA**GGATCC**CTTCATCGACGCCTTCAACT		
**NsiI**-orf9	TATA**ATGCAT**TGACGACAGTTGCCTTCTTG	2501-bp upstream flanking region for *orf9* deletion	**pDorf9** (Δorf9)
**SpeI**-orf9	TATA**ACTAGT**CAGTTCCACCAACTCCACCT		
**EcoRV**-orf9	TATA**GATATC**ACATGTCGATCCCGAAGAGT	2495-bp downstream flanking region for *orf9* deletion	
**BglII**-orf9	TATA**AGATCT**CCTCCTACGAAGGGGAGTTC		
**SpeI**-orf16	TATA**ACTAGT**CCCTTCTTGAAAGGACCACA	2659-bp upstream flanking region for *orf16* deletion	**pDorf16** (Δorf16-luxR)
**NsiI**-orf16	TATA**ATGCAT**GAGTCATGATCACGCTCCAG		
**BamHI**-orf16	TATA**GGATCC**GTCCTCGCGAAGCTACAGTT	2592-bp downstream flanking region for *orf16* deletion	
**EcoRV**-orf16	TATA**GATATC**ATCTCCGTGAGAGTGGCATC		
**SpeI**-orf20	TATA**ACTAGT**TCGCGTACAACATCATCGAG	2510-bp upstream flanking region for *orf20* deletion	**pDorf20** (Δorf20-marR)
**NsiI**-orf20	TATA**ATGCAT** GTGCTGGGTGACGTCCTT		
**BamHI**-orf20	TATA**GGATCC**CGTCTACGCCGTCGAACAG	2353-bp downstream flanking region for *orf20* deletion	
**EcoRV**-orf20	TATA**GATATC**AAGTGGTTGCCGATGAAGAG		

Table S1. Primers and plasmids used in this work (continued)

Primer	Sequence (5'-3')	PCR product and purpose	Plasmid (related strain)
		**Gene mutation**	
**SpeI**-orf29	TATA**ACTAGT**CCTGGAGATATCCCTCACCA	2392-bp upstream flanking region for *orf29* deletion	**pDorf29** (Δorf29-gntR)
**NsiI**-orf29	TATA**ATGCAT**GCTGGGACAGCTGGAAATAC		
**BglII-**orf29	TATA**AGATCT**GTTACGCCTTCGAGTTCCAG	2609-bp downstream flanking region for *orf29* deletion	
**EcoRV**-orf29	TATA**GATATC**CTGCGATCGAGAAGAAAAGG		
**SpeI**-orf31	TATA**ACTAGT**GTCTCCAGCGTGAGACCACT	2345-bp upstream flanking region for *orf31* deletion	**pDorf31** (Δorf31)
**NsiI**-orf31	TATA**ATGCAT**GTGAAGGGGCGGTAGTCG		
**BglII**-orf31	TATA**AGATCT**ACGACGAGTTCAAGGCGTAT	2316-bp downstream flanking region for *orf31* deletion	
**EcoRV**-orf31	TATA**GATATC**ATGACGCAGAGCACCTTCTT		
**SpeI**-M	TATA**ACTAGT**CCTCGCGTACGTCATCTACA	2506-bp upstream flanking region for *jomM* deletion	**pDjomM** (ΔjomM)
**NsiI**-M	TATA**ATGCAT**CGTGGTTGTACACCTCGGATA		
**BglII**-M	TATA**AGATCT**GACTACCTCCCCACACTGGA	2751-bp downstream flanking region for *jomM* deletion	
**EcoRV**-M	TATA**GATATC**CGGGAGCTGAACTGTAGCTT		
		**Gene expression**	
**EcoRV**-araC	TATA**GATATC**GTGGTGGCGAGCCCTAAG	917-bp fragment containing *orf8* (-31 bp from start codon) for gene overexpression	**pSAraC** (G- pSAraC)
**BamHI**-araC	TATA**GGATCC**CTCTGTGGGTCTTCCGTCC		
**EcoRV**-luxR	TATA**GATATC**CTACCGCGCCGGCCGCT	2376-bp fragment containing *orf16* (-100 bp from start codon) for gene overexpression	**pSLuxR** (G- pSLuxR)
**XbaI**-luxR	TATA**TCTAGA**GTGTCCCTTCCGGAGCAAAG		
**EcoRV**-marR	TATA**GATATC**GTCATTCCTCCAGGGCCG	547-bp fragment containing *orf20* (-84 bp from start codon) for gene overexpression	**pSMarR** (G- pSMarR)
**BamHI**-marR	TATA**GGATCC**TGGGGTCTTCCTCACTCCTT		
**EcoRV**-gntR	TATA**GATATC**CAGGCGGACGGAATGACC	888-bp fragment containing *orf29* (-60 bp from start codon) for gene over-expression	**pSGntR** (G- pSGntR)
**BamHI**-gntR	TATA**GGATCC**TGTCCGGACAATGTGACCTC		
**SmaI**-jomM	ATAT**CCCGGG**CTGTGGGTCAGCTGGCGG	1035-bp fragment containing *jomM* (-38 bp from start codon) for mutant complementation	**pSEJomM** (ΔM-pSEJomM)
**BamHI**-jomM	ATAT**GGATCC**CCGAAGAGCTTGGTCAACGA		
**EcoRI**-KanR	TATA**GAATTC**GAAATCTCGTGATGGCAGGT	930-bp fragment containing gene *aph(3) II* (-86 bp from start codon) for pSETk construction	**pSETk** (G- pSETk)
**EcoRV**-KanR	TATA**GATATC**TTTCTTGCCGCCAAGGATCT		

Table S1. Primers and plasmids used in this work (continued)

Primer	Sequence (5'-3')	PCR product and purpose	Plasmid (related strain)
		**JAs BGC heterologous expression**	
XhoI-2TAR	TATA**CTCGAG**CTTAGGGCTCGCCACCAC	1081-bp arm flanking JAs BGC (left) for capture by TAR cloning	**pCL2-CAP** (Sc-pJATAR and Sa-pJATAR)
NsiI-U2TAR	TATA**ATGCAT**AGCTCATGGGACTGAACACC		
NsiI-L2TAR	TATA**ATGCAT**GAGCGTGATCATGACGAGGT	945-bp arm flanking JAs BGC (right) for capture by TAR cloning	
SpeI-2TAR	TATA**ACTAGT**TGGTGTTGATCACCCACAAT		
dApra-pErm	caggttctttagggcagggcgaccgtcctctcgggtcgcattTTTTCTACGGGGTCTGACGC	Amplification of a *aac*(*3*)*IV*-*ermE**p* assembled fragment to be inserted upstream of *jomP1* at pJATAR	**pNTARe** (Sc-pJATARe and Sa-pJATARe)
dApra	TTTTCTACGGGGTCTGACGC		
NsiI-rvApra	TATA**ATGCAT**TCGTTAGTCGGAGGCCAAAC		
dNsiI-pErm	TATA**ATGCAT**GTAAAACGACGGCCAGTGC		
rvApra-pErm	tcaccggcggtcttcattcaacatccccaatttcatgaagtgTACGAATTCGATATCGCGCG		
rvpErm	TACGAATTCGATATCGCGCG		
		**Mutant confirmation**	
apraI	TGAGAAGCTGACCGATGAGCTC	reverse primer annealing *aac(3)IV*	
apraII	GAGCTCATCGGTCAGCTTCTCA	forward primer annealing *aac(3)IV*	
dcfErm	CGGTTGGTAGGATCCAGACCTG	Confirmation of *ermE**p* insertion upstream of *jomP1*	
rvcfErm	GAACCTGTGAGGCCAGCGA		
cfP1up	GGACTGATGAAGGTGGTGCTGT	used with apraI for confirmation of *jomP1* interruption with pIjomP1	
cfP1dw	CAGGTGGAAGTAGTCGTCCTCG	used with apraII for confirmation of *jomP1* interruption with pIjomP1	
cfNup	TCCTCGTACCACTGGGAAGAGA	used with apraI for upstream confirmation of *jomN* interruption with pIjomN	
cfNdw	GAGAACTCCACCTGCCATTCCT	used with apraII for downstream confirmation of *jomN* interruption with pIjomN	
cf8up	CGGTTGTTGGGCGACTTGATC	used with apraI for upstream confirmation of *orf8* deletion	
cf8dw	CTTCAGGTACGGGGTGTCGATC	used with apraII for downstream confirmation of *orf8* deletion	
cf9up	CTTCGATCCTGGTGATCCGCC	used with apraI for upstream confirmation of *orf9* deletion	
cf9dw	TCCCTTGATGCCAGCTGTAGAC	used with apraII for downstream confirmation of *orf9* deletion	

Table S1. Primers and plasmids used in this work (continued)

Primer	Sequence (5'-3')	PCR product and purpose
		**Mutant confirmation**
cf16up	AGGAATGGCAGGTGGAGTTCTC	used with apraI for upstream confirmation of *orf16* deletion
cf16dw	AGCACAAAAACGGAGCAGGTTG	used with apraII for downstream confirmation of *orf16* deletion
cf20up	CCATGAACTCGATGGGCAGGTA	used with apraI for upstream confirmation of *orf20* deletion
cf20dw	TCTCGTCGGTGATGTGCTTGAT	used with apraII for downstream confirmation of *orf20* deletion
cf29up	GAAAATGCCGTATCCGCCGTAG	used with apraI for upstream confirmation of *orf29* deletion
cf29dw	CCTCCATATCGGCGATGGTCTT	used with apraII for downstream confirmation of *orf29* deletion
cf31up	CGGATACCGTGGTGCCATAGG	used with apraI for upstream confirmation of *orf31* deletion
cf31dw	CTCGATCGAGGACTGCGAGTTC	used with apraII for downstream confirmation of *orf31* deletion
cfMup	ATCCTGCTGGACGAACAGACC	used with apraI for upstream confirmation of *jomM* deletion
cfMdw	CTCTTCATCGGCAACCACTT	used with apraII for downstream confirmation of *jomM* deletion

Figure S27. Confirmation by PCR amplification of *S. caniferus* GUA-06-05-006A derivative strains generated in this work. Recombinant strain (RS) G-pErm* and mutant strains (M) were subjected to colony PCR with the indicated primer pairs (see table S1). In all cases a negative PCR control (WT) consisting in wild type *S. caniferus* GUA-06-05-006A DNA was processed. Band sizes were compared with 0.1 μg of lambda/PstI DNA marker.

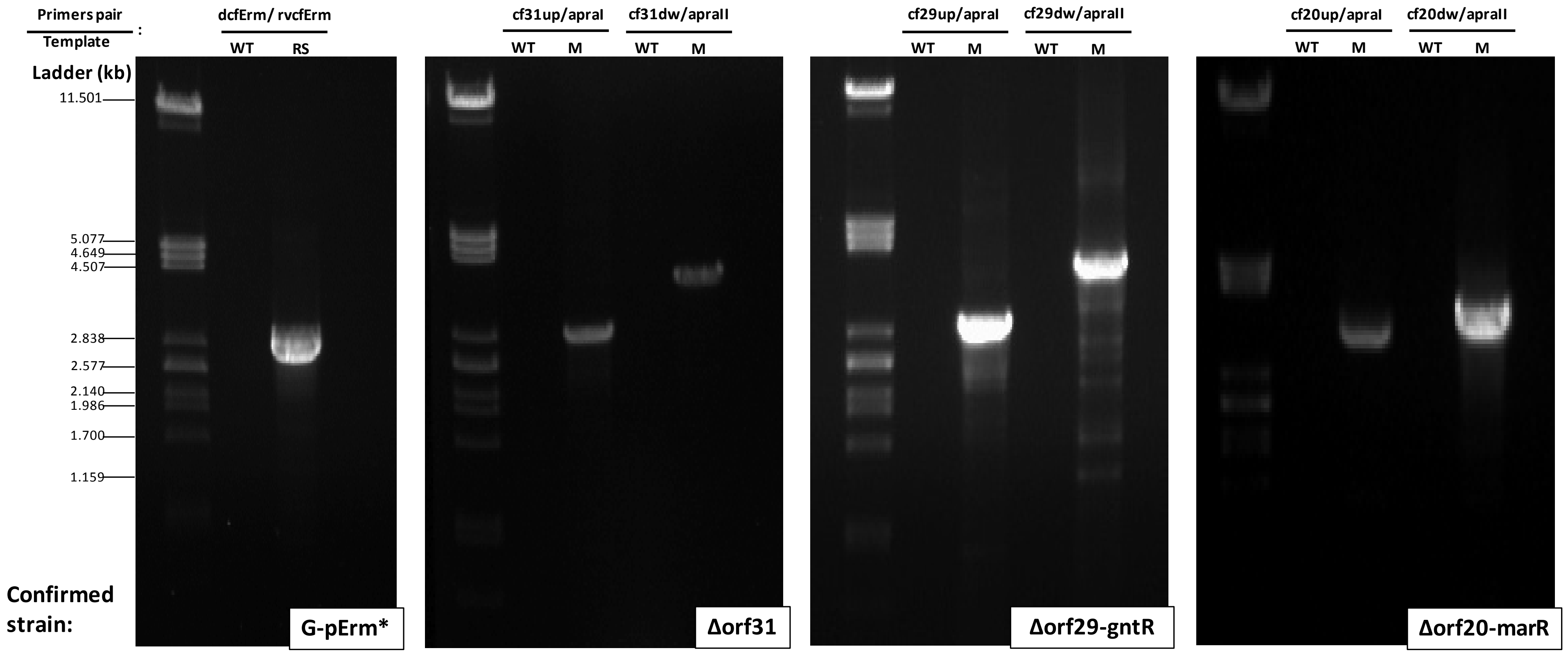

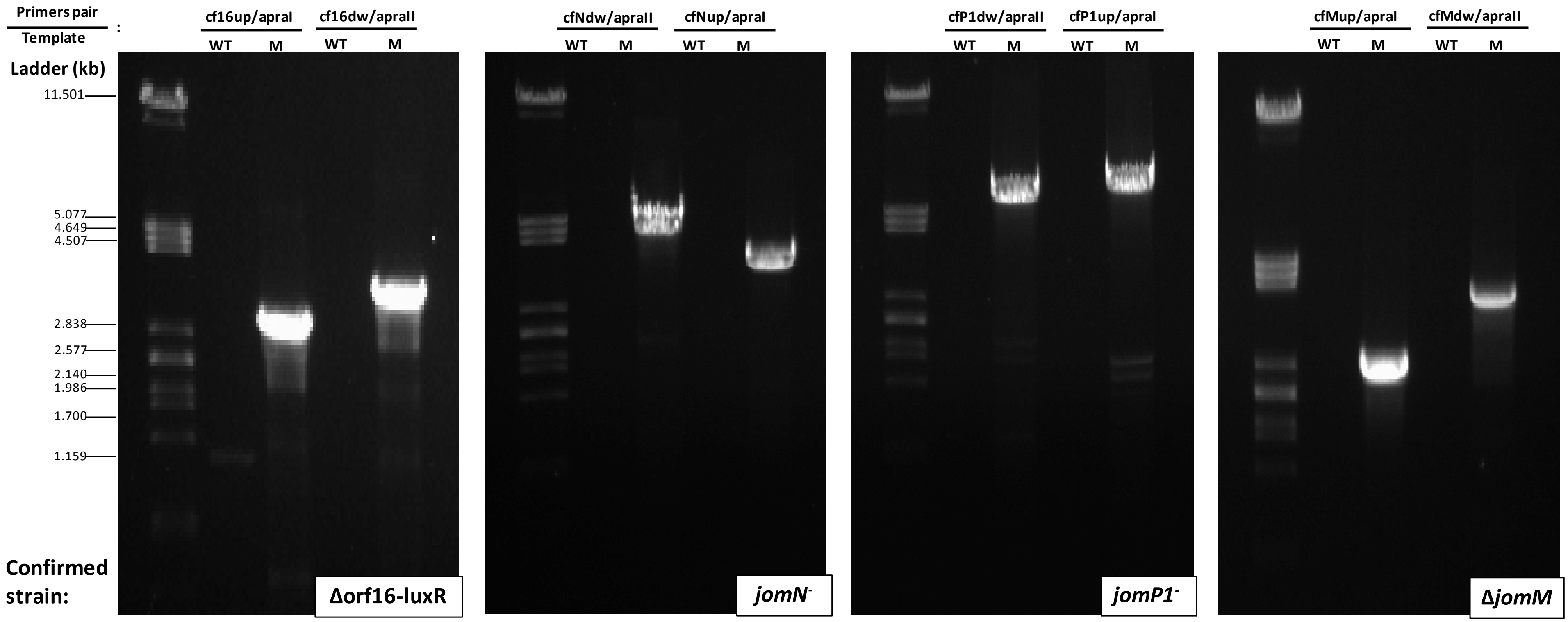
Primers pair
Template :
cf16up/apraI
cf16dw/apraII
cfNdw/apraII
cfNup/apraI
cfP1dw/apraII
cfP1up/apraI
cfMup/apraI
cfMdw/apraII
WT M WT M
Ladder (kb)
11.501
5.077
4.649
4.507
2.838
2.577
2.140
1.986
1.700
1.159
Confirmed strain:
Δorf16-luxR
jomN-
jomP1-
ΔjomM

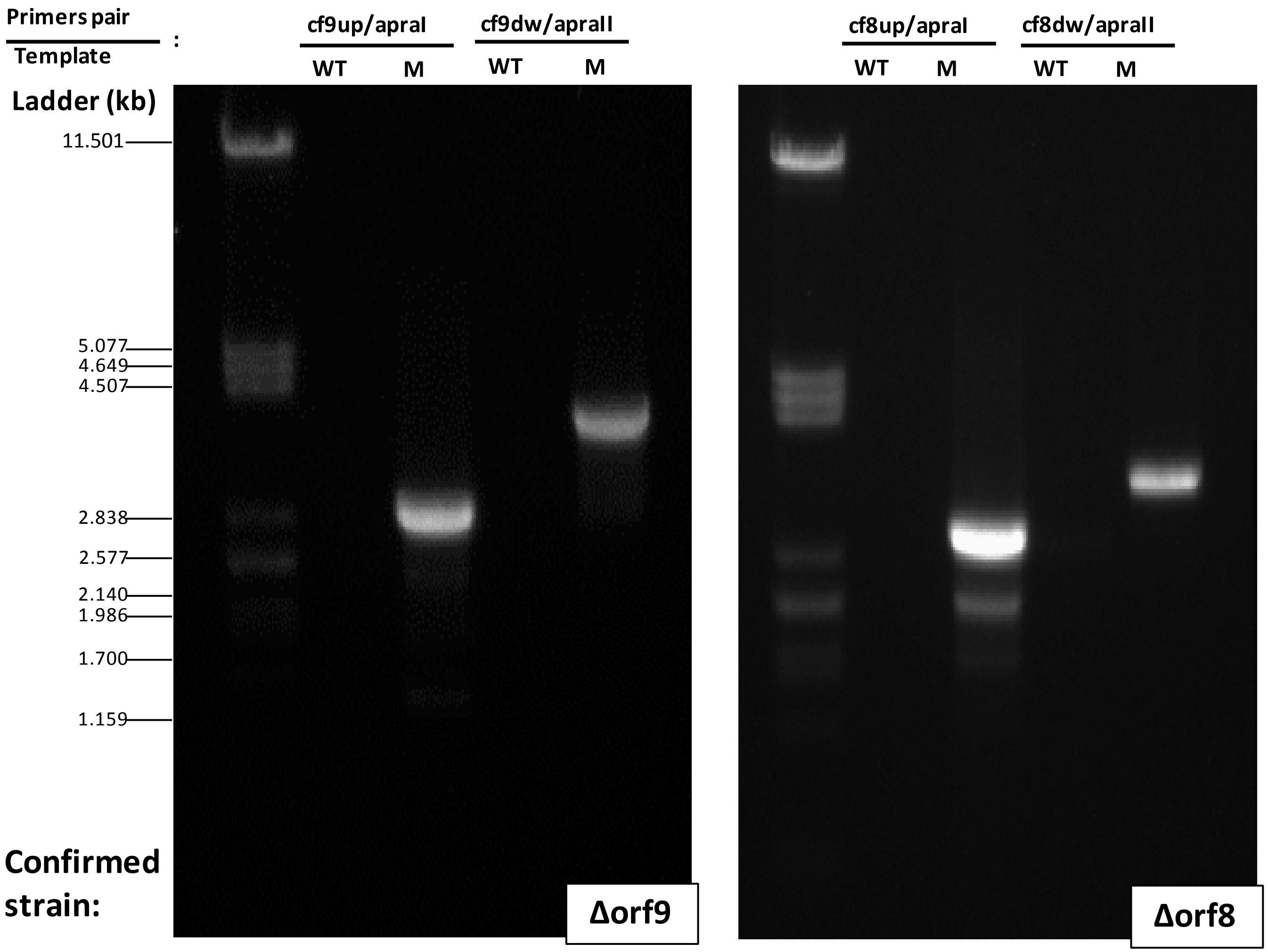

Primers pair
Template
:
cf9up/apraI
cf9dw/apraII
cf8up/apraI
cf8dw/apraII
WT
M
WT
M
WT
M
WT
M
Ladder (kb)
11.501
5.077
4.649
4.507
2.838
2.577
2.140
1.986
1.700
1.159
Confirmed strain:
Δorf9
Δorf8
